# Variation in Grain Zinc and Iron Concentrations, Grain Yield and Associated Traits of Biofortified Bread Wheat Genotypes in Nepal

**DOI:** 10.3389/fpls.2022.881965

**Published:** 2022-06-13

**Authors:** Dhruba Bahadur Thapa, Mahesh Subedi, Rajendra Prasad Yadav, Bishnu Prasad Joshi, Bhim Nath Adhikari, Keshav Prasad Shrestha, Prem Bahadur Magar, Khem Raj Pant, Suk Bahadur Gurung, Sapana Ghimire, Nutan Raj Gautam, Nav Raj Acharya, Manoj Sapkota, Vinod Kumar Mishra, Arun Kumar Joshi, Ravi Prakash Singh, Velu Govindan

**Affiliations:** ^1^Nepal Agricultural Research Council (NARC), Kathmandu, Nepal; ^2^Institute of Plant Breeding, Genetics, and Genomics, University of Georgia, Athens, GA, United States; ^3^Department of Genetics and Plant Breeding, Banaras Hindu University (BHU), Varanasi, India; ^4^International Maize and Wheat Improvement Center (CIMMYT), New Delhi, India; ^5^International Maize and Wheat Improvement Center (CIMMYT), Texcoco, Mexico

**Keywords:** biofortified wheat, HarvestPlus, grain yield, grain iron concentration, grain zinc concentration

## Abstract

Wheat (*Triticum aestivum* L.) is one of the major staples in Nepal providing the bulk of food calories and at least 30% of Fe and Zn intake and 20% of dietary energy and protein consumption; thus, it is essential to improve its nutritional quality. To select high-yielding genotypes with elevated grain zinc and iron concentration, the sixth, seventh, eighth, and ninth HarvestPlus Yield Trials (HPYTs) were conducted across diverse locations in Nepal for four consecutive years: 2015–16, 2016–17, 2017–18, and 2018–19, using 47 biofortified and 3 non-biofortified CIMMYT-bred, bread wheat genotypes: Baj#1, Kachu#1, and WK1204 (local check). Genotypic and spatial variations were found in agro-morphological traits; grain yield and its components; and the grain zinc and iron concentration of tested genotypes. Grain zinc concentration was highest in Khumaltar and lowest in Kabre. Likewise, grain iron concentration was highest in Doti and lowest in Surkhet. Most of the biofortified genotypes were superior for grain yield and for grain zinc and iron concentration to the non-biofortified checks. Combined analyses across environments showed moderate to high heritability for both Zn (0.48–0.81) and Fe (0.46–0.79) except a low heritability for Fe observed for 7th HPYT (0.15). Grain yield was positively correlated with the number of tillers per m^2^, while negatively correlated with days to heading and maturity, grain iron, grain weight per spike, and thousand grain weight. The grain zinc and iron concentration were positively correlated, suggesting that the simultaneous improvement of both micronutrients is possible through wheat breeding. Extensive testing of CIMMYT derived high Zn wheat lines in Nepal led to the release of five biofortified wheat varieties in 2020 with superior yield, better disease resistance, and 30–40% increased grain Zn and adaptable to a range of wheat growing regions in the country – from the hotter lowland, or Terai, regions to the dry mid- and high-elevation areas.

## Introduction

Bread wheat (*Triticum aestivum* L.) occupies the third position after rice and maize in terms of production and the second position after rice in terms of consumption in Nepal. In 2019–20, wheat was grown on 707,505 ha, with production and productivity of 2,185,288 mt and 2.92 mt/ha, respectively (MOALD, 2019). More than 1 million farmers collectively produce around two million tons of wheat each year, which is consumed as *chapatis, rotis, momo* dumplings, and noodles made from the grain. Wheat grain is known to be low in essential micronutrients, contributing to poor nutrition (CIMMYT, 2021). Despite the improvement in food availability, malnutrition is still a major challenge in developing countries like Nepal. Micronutrient malnutrition is severe in the Nepalese population. There is an 8.1% prevalence of undernourishment, 37.1% of stunting, 11.3% of wasting, and 30.1% of people are underweight (FAO, 2017). About 41% of women of reproductive age and 53% of children in Nepal were found to be anemic (Ministry of Health, 2017). More than three-quarters of apparently healthy women were found to be zinc deficient in Bhaktapur, Nepal (Chandyo et al., 2009).

In countries with a high incidence of micronutrient deficiencies, cereal-based foods represent the largest proportion of the daily diet (Bouis and Welch, 2010). About 2–3% of the GDP (US$ 250–375 million) is lost every year in Nepal on account of vitamin and mineral deficiencies (World Bank, 2012). Trace elements like zinc and iron have been shown to play key roles in supporting the human immune system and reducing the risk of infections (Calder, 2020). Moreover, intellectual ability is affected by a deficiency of iron and zinc (Mari et al., 1995; Falkingham et al., 2010). The diets of over two-thirds of the world's population reportedly lack one or more essential mineral elements: iron (Fe), zinc (Zn), copper (Cu), calcium (Ca), magnesium (Mg), iodine (I), and selenium (Se), and this can be remedied through biofortification (White and Broadley, 2009). To overcome micronutrient malnutrition, the development of biofortified crop varieties with the ability to harvest more essential nutrients from the soil is a low-cost, sustainable solution (Bouis and Welch, 2010; Bouis et al., 2011).

Cereals—mainly wheat, rice, and maize—are a major source of dietary energy, protein, and micronutrients for the resource poor people in Nepal. Wheat is a high-potential crop that can assist in reducing micronutrient-related malnutrition; this reduction can be enhanced through the integration of agronomic practices and the delivery of genetically improved micronutrient-rich wheat varieties that have been developed by breeding common cultivars with wild wheat that is rich in zinc and iron. In respect of wheat, wild wheat varieties *Triticum dicoccoides, Aegilops tauschii, Triticum monococcum, and Triticum boeticum* are among the most promising sources of high Fe and Zn, with high genetic variation for micronutrients, whereas improved wheat is low in micronutrients (Cakmak et al., 2000, 2004). Therefore, the biofortification breeding program at CIMMYT introgressed high Zn and Fe progenitors into elite wheat lines and successfully introduced novel QTLs for grain Zn and Fe in elite wheat lines with better yield potential, stress tolerance, and elevated grain Zn/Fe concentration (Velu et al., 2014).

Wheat breeding in Nepal has been mainly focused on grain yield, but the nutritional quality of wheat is a less-explored aspect. As the growing global momentum on the nutrition security, breeding for better nutrition has gained momentum in Nepal as well. Since 1960, Nepal has released 52 bread wheat and 2 durum wheat varieties. Beginning in 2015, the Nepal Agricultural Research Council (NARC) has introduced and initiated testing CIMMYT-developed biofortified wheat genotypes in different locations in the country. The fast-track testing, identification, release, multiplication, and dissemination of varieties have now been identified as one of the main options available for Nepalese breeders and agricultural scientists to combat hidden hunger. In this article, we evaluate different biofortified wheat lines developed by CIMMYT for zinc concentration, iron concentration, and several agronomic traits for 4 years at different national research centers, with the aim of selecting competitive national and region-specific best-performing biofortified wheat lines.

## Materials and Methods

### Plant Material

This study includes elite high Zn lines developed at CIMMYT, Mexico, and introduced to Nepal for testing and advancement. This includes four cycles of HarvestPlus Yield Trial (HPYT) sets: the 6th, 7th, 8th, and 9th HPYTs from CIMMYT Mexico (Supplementary Tables 1–4), were tested for 4 years under irrigated conditions at five locations in Nepal. This study includes wheat genotypes included in the 6th HPYT to 9th HPYT distributed and each HPYT trial consists of two replicates of 50 entries arranged in an alpha-lattice design. Each HPYT includes 47 new bread wheat lines, 2 CIMMYT checks (Baj#1, an early-maturity check and Kachu#1 or Mayil, a normal-maturity check), and WK1204 as local check.

### Experimental Sites

The HPYTs were grown across environments of Nepal including Doti, Kabre, Khumaltar, Bhairahawa, Nepalgunj, and Surkhet. During the 2015–16 crop cycle, three sets of the 6th HPYT were tested at the Agriculture Research Station, Dasharathpur (Surkhet), the Hill Crops Research Program, Kabre (Dolakha), and the Agriculture Botany Division, Khumaltar (Lalitpur). Similarly, in 2016–17, four sets of the 7th HPYT were tested at the Regional Agriculture Research Center, Dipayal (Doti), the Hill Crops Research Program, Kabre (Dolakha), the Agriculture Botany Division, Khumaltar (Lalitpur), and the Agriculture Research Center, Dasharathpur (Surkhet). During 2017–18, five sets of the 8th HPYT were tested at the National Wheat Research Program, Bhairahawa, the Regional Agriculture Research Center, Dipayal, (Doti), the Regional Agricultural Research Station, Khajura (Nepalgunj), the Hill Crops Research Program, Kabre (Dolakha), and the Agriculture Botany Division, Khumaltar (Lalitpur). In 2018–19, five sets of the 9th HPYT were tested at the National Wheat Research Program, Bhairahawa, the Regional Agriculture Research Center, Dipayal, (Doti), the Hill Crops Research Program, Kabre (Dolakha), the Agriculture Botany Division, Khumaltar (Lalitpur) and the Agriculture Research Center, Dasharathpur (Surkhet).

### Trial Management and Data Collection

Each trial was grown in a 5 m^2^ plot and fertilizer was applied at the rate of 120:60:60 kilograms of nitrogen, phosphorus, and potash per hectare. The full dose of phosphorus and potash and half the dose of nitrogen were applied at the time of preparing the land for sowing. The remaining half dose of nitrogen was divided into two parts: one part (i.e., 30 kg) was applied at 25 days from seed emergence and another 30 kg was applied during the crop booting stage. The genotypes were evaluated for grain yield and related traits and zinc and iron concentration. Grain samples were analyzed with a bench-top, non-destructive, energy-dispersive X-ray fluorescence spectrometry (EDXRF) instrument (model x-supreme8000, Oxford Instruments plc, Abingdon, UK) that was standardized for the high-throughput screening of grain iron (GZnC) and grain zinc (GFeC) (unit: mg/kg) concentrations in whole grain wheat (Paltridge et al., 2012). Days to heading were recorded as the number of days to when 50% of total plants in a plot had extended a spike from the leaf sheath. Days to maturity were recorded when 80% of the plants in a plot had peduncles that had turned from green to golden yellow. Plant height was measured as the length from ground level to the apex of the spike excluding awns. For grain number per spike (GNS) and grain weight per spike (GWS), we sampled 10 spikes per plot and threshed it and counted manually and weighed, and then averaged it for GNS and GWS. The number of total heads per square meter was assessed by measuring the number of tillers counted and converted into the number of heads per square meter. Spike length was measured on a representative spike within the plot as the length from the base to the tip of a spike excluding awns.

### Statistical Analysis

The datasets were analyzed using Best Linear Unbiased Prediction (BLUP) based on the variance component in the mixed model, in which genotype effects, block, and location were all set as random. Phenotypic correlation matrix and cluster analysis based on the BLUP values of the studied traits of the 50 genotypes combined over locations were analyzed in META-R and RStudio 3.4.3 (Schwarzer et al., 2015; RStudio Team, 2020). The cluster dendrograms were constructed using “ggtree” package in R (Yu et al., 2017). Broad-sense heritability (H^2^) for all the studied traits was computed according to Allard (1960). Expected genetic advance (GA) assuming a selection intensity of 5% was computed following the formula developed by Allard (1960). Correlation analysis was performed by calculating Pearson's correlation coefficient using a ggcorrplot package in R (Kassambara and Kassambara, 2019).

## Results

### Descriptive Statistics

#### 6th HPYT 2015–16

In 2015–16, the genotypes of 6th HPYT tested at Surkhet, Kabre, and Khumaltar varied significantly for the following studied traits: days to heading and maturity; plant height; thousand grain weight (Table 1). All these traits including grain yield were found to be significantly different across the locations. The shortest period to heading was found at Surkhet and the longest at Khumaltar. Similarly, the average number of days to maturity was found to be highest at Khumaltar. A similar trend was found for plant height. Thousand grain weight was similar at Surkhet and Khumaltar but greater at Kabre. The overall mean for thousand grain weight in the tested genotypes was 47.6 g across the locations; the highest value was found at Khumaltar (50.6 g) and the lowest at Surkhet (46.0 g). The highest grain yield (4.51 t/ha), grain zinc concentration (48.78 mg/kg), and grain iron concentration (46.77 mg/kg) were found in Khumaltar, while Surkhet had the lowest grain zinc (20.26 mg/kg) and grain iron concentrations (34.64 mg/kg), but the grain yield was almost double that of Kabre. Among the tested genotypes, entry 437 recorded the maximum grain yield across locations (3,423 kg/ha) and entry 404 [CROC_1/AE.SQUARROSA(210)//INQALAB 91^*^2/KUKUNA/3/PBW343^*^2/KUKUNA] had the maximum concentration of zinc (36.3 mg/kg) as well as iron (44.8 mg/kg) in grains, while the non-biofortified check variety (entry 401, WK1204) recorded the lowest zinc concentration (33.3 mg/kg). High heritability was found in days to heading (0.68) and thousand grain weight (0.88). Moderate heritability was found in days to maturity (0.54), plant height (0.48), grain yield (0.3), grain iron concentration (0.46), and grain zinc concentration (0.58). High genetic advance as a percentage of mean was found in grain zinc concentration (13.6%) and thousand grain weight (13%) (Table 2).

**Table 1:** Top five high yielding lines from 6th, 7th, 8th and 9th HarvestPlus Yield Trial during 2015–16 to 2018–19 along with the mean value and ANOVA p-level statistics for corresponding traits calculated across all tested genotypes and locations in the respective years.

**Entry no**.	**DTH**	**DTM**	**Pht** **(cm)**	**Tillers**	**GNS**	**GWS** **(gm)**	**TGW** **(gm)**	**GY** **(kg/ha)**	**GrainFe** **(mg/kg)**	**GrainZn** **(mg/kg)**
**6th HPYT**										
431	111	165	90				53.8	3,420	41.9	34.3
433	111	166	91				54.8	3,370	41.4	33.4
437	109	165	87				49.5	3,423	40.8	33.6
443	110	166	90				47.2	3,407	42.6	33.4
446	111	166	88				46.3	3,391	41.4	34.7
447	110	163	91				47.7	3,382	42.7	35.3
Kabre	108	158	81				50.5	1,887	43.7	34.2
Khumaltar	131	173	94				46.1	4,506	46.7	48.7
Surkhet	91	X	92				46.1	3,560	34.7	20.3
Mean	110	165	89				47.6	3,318	41.7	34.4
p-level G	0.000	0.000	0.000				0.000	0.475	0.062	0.255
p-level Loc	0.000	0.000	0.000				0.000	0.000	0.000	0.000
p-level G X Loc	0.004	0.000	0.000				0.994	0.155	0.990	0.999
**7th HPYT**										
402	100	146	88	277	52	2.7	48.8	4,048	43.8	33.3
411	107	152	88	261	54	2.9	53.5	4,036	44.7	32.7
429	103	152	90	271	50	2.7	49.6	4,026	45.7	35.5
441	101	149	92	252	53	2.8	52.3	4,022	44.2	34.5
446	106	152	91	279	55	2.9	52.1	4,016	42.1	32.9
Doti	93	134	95	X	X	X	46.3	2,616.5	53.7	35.8
Kabre	111	169	78	222.7	X	X	53	3,189.8	44.6	31.5
Khumaltar	126	174	104	310.6	58.5	3.1	52.9	6,527.7	40.8	43.9
Surkhet	84	123	87	254.6	46.4	2.5	54.2	2,809.4	37.5	23.5
Mean	104	150	91	262.6	52.5	2.8	51.6	3,785.8	44.1	33.7
p-level G	0.000	0.000	0.000	0.003	0.004	0.031	0.000	0.004	0.025	0.016
p-level Loc	0.000	0.000	0.000	0.000	0.000	0.000	0.000	0.000	0.000	0.000
p-level G X Loc	0.007	0.000	0.820	0.000	0.169	0.132	0.086	0.000	0.151	0.573
**8th HPYT**										
402	103	145	84	227	53	X	47.8	3,606	41.4	28.8
405	105	147	80	218	53	X	46.4	3,803	39.8	28.7
412	105	148	84	202	53	X	51.6	3,617	38.7	31
416	104	148	87	204	53	X	49.7	3,863	41.4	30.2
429	103	147	88	209	53	X	51.9	3,670	40	28.8
Doti	96	136	84	192	47	X	47.7	3,801	48.1	26
Kabre	108	150	68	196	0	X	47.5	2,113	36.3	20.1
Khumaltar	114	161	98	206	59	X	48.9	3,898	40.1	42
Bhairahawa	X	X	X	X	X	X	X	X	32.8	29.2
Nepalgunj	X	X	X	X	X	X	X	X	42.5	29.4
Mean	106	149	83	9.4	198	X	48	3,271	40	29.3
p-level G	0.000	0.000	0.802	0.029	0.006	X	0.000	0.001	0.000	0.002
p-level Loc	0.000	0.000	0.000	0.000	0.145	X	0.006	0.000	0.000	0.000
p-level G X Loc	0.001	0.176	0.916	0.987	0.810		0.000	0.000	0.225	0.034
**9th HPYT**										
406	103	145	87	364	59	2.56	43.5	4,716	33.3	28
421	100	144	87	363	55	2.43	43.1	4,724	32.1	29.2
431	103	146	85	365	57	2.48	43.1	4,757	31.1	28.6
440	101	144	88	362	55	2.57	44.4	4,775	32.2	28.9
450	100	145	90	367	56	2.54	44	4,757	31.3	30.7
Bhairahawa	86	119	92				41.5	4,169.7	34	35.8
Doti	101	143	89				42	4,207.1	31.3	19.1
Kabre	105	149	72	254	48.2	2.39	49.2	3,555.6	34.1	28.6
Khumaltar	121	165	103	464	63.1	2.69	43	6,239.6	34.6	48.1
Surkhet	93		77				44.4	4,829.4	29.7	19.5
Mean	101.1	144	86.6	358.8	55.7	2.54	44	4,600.3	32.7	30.2
p-level G	0.000	0.000	0.000	0.368	0.002	0.081	0.199	0.222	0.000	0.000
p-level Loc	0.000	0.000	0.000	0.000	0.000	0.000	0.000	0.000	0.000	0.000
p-level G X Loc	0.127	0.000	0.985	0.553	0.156	0.067	0.447	0.298	0.851	0.105

*DTH, days to heading; DTM, days to maturity; Pht, plant height; TGW, thousand grain weight; GY, grain yield; GrainFe, grain iron concentration; GrainZn, grain zinc concentration*.

**Table 2:** Heritability and genetic advance of studied traits of HPYTs using BLUP values, 2015/16–2018/19 seasons.

**Parameter**	**DTH**	**DTM**	**Pht**	**TGW**	**GY**	**GrainFe**	**GrainZn**
**6**^**th**^ **HPYT-2015/16**							
Heritability	0.68	0.54	0.48	0.88	0.3	0.46	0.58
GA% of mean	3.1	1.8	5.9	13	7.2	6.1	13.6
**7**^**th**^ **HPYT-2016/17**							
Heritability	0.71	0.43	0.67	0.67	0.2	0.15	0.48
GA% of mean	23	15	17	14	21	5	19
**8**^**th**^ **HPYT-2017/18**							
Heritability	0.64	0.71	0.4	0.72	0.57	0.79	0.52
GA% of Mean	7	4	5	13	26	25	16
**9**^**th**^ **HPYT-2018/2019**							
Heritability	0.85	0.65	0.76	0.21	0.33	0.56	0.81
GA % of mean	24	18	25	7	20	50	19

*DTH, days to heading; DTM, days to maturity; Pht, plant height; TGW, thousand grain weight; GY, grain yield; GrainFe, grain iron concentration; GrainZn, grain zinc concentration*.

#### 7th HPYT 2016–17

The genotypes of 7th HPYT tested at Doti, Kabre, and Khumaltar were found to have highly significant differences across the locations for all the studied traits except grain Fe content (Table 1). Overall, the longest periods for days to heading and maturity were found at Khumaltar and the shortest at Surkhet. On average, plants were found shortest in height at Kabre and tallest under conditions at Khumaltar. Thousand grain weights were very similar at Kabre and Khumaltar but lower at Doti. The highest grain yield (6.53 t/ha) and highest grain zinc concentration (43.9 mg/kg) were found at Khumaltar, while the grain iron concentration (53.6 mg/kg) was higher at Doti than at Kabre and Khumaltar. The non-biofortified check variety, Baj#1 (entry 402) recorded the maximum grain yield (4,048 kg/ha), while entry 404 showed the highest grain Zn (38.1 mg/kg). High heritability was found in days to heading (0.71), plant height (0.67), thousand grain weight (0.67); moderate heritability was found in days to maturity (0.43) and grain zinc concentration (0.48), while low heritability was found in grain yield (0.2) and grain iron concentration (0.15). High genetic advance as a percentage of mean was found for grain yield (21%), moderate advance for grain zinc concentration (19%), and low advance for grain iron concentration (5%) (Table 2).

#### 8th HPYT 2017–18

In 2017–18, 8th HPYT tested at Bhairahawa, Doti, Kabre, and Khumaltar were found to have highly significant differences across all the locations for all the studied traits except plant height and thousand grain weight. Overall, the length of time to heading and maturity for the tested genotypes was longest at Khumaltar and shortest at Doti. Mean plant height was shortest at Kabre and tallest at Khumaltar. Thousand grain weight was heaviest at Khumaltar (48.9 gm) by a significant amount. Grain yield was highest at Khumaltar (3.9 t/ha), followed by Doti (3.8 t/ha), and finally Kabre (2.1 t/ha). Grain iron concentration was highest at Doti (48.1 mg/kg) and was lowest at Bhairahawa (32.8 mg/kg). Grain zinc concentration was highest at Khumaltar (42.1 mg/kg) and lowest at Kabre (20.1 mg/kg). Entry 417 recorded the highest grain zinc (31.8 mg/kg) and iron (49.2 mg/kg) concentrations among the tested genotypes. Conversely, it produced the lowest grain yield (2,829 kg/ha). High heritability was found in days to heading (0.64), days to maturity (0.71), thousand grain weight (0.72), and grain iron concentration (0.79). Moderate heritability was found in grain yield (0.57), grain zinc concentration (0.52), and plant height (0.4). Genetic advance as a percentage of mean was found high in grain yield (26%), grain iron concentration (25%); moderate genetic advance was found in grain zinc concentration (16%) and thousand grain weight (13%) (Table 2).

#### 9th HPYT 2018–19

In 2018–19, genotypes tested in 9th HPYT at Bhairahawa, Doti, Surkhet, Kabre, and Khumaltar were found to be different at highly significant levels for days to heading and maturity, plant height, and concentrations of grain iron and zinc, and at non-significant levels for thousand grain weight and grain yield. All the studied traits of the tested genotypes were found to have highly significant differences according to location (Table 1). The overall length of time to heading and maturity was longest at Khumaltar and shortest at Bhairahawa. Plant height was shortest at Kabre and tallest at Khumaltar. Thousand grain weight was heaviest at Kabre, but grain size was similar at the other locations. The highest grain yield was at Khumaltar, and the lowest was at Kabre. The highest grain iron (40.1 mg/kg) and zinc concentrations (42.08 mg/kg) were found at Khumaltar Among the tested genotypes, Zinc Shakti (entry 404) recorded the maximum grain zinc (33.5 mg/kg) and iron (36.6 mg/kg) concentration, while entry 440 recorded the maximum grain yield (4,775 kg/ha) but was at par in terms of grain zinc (28.9 mg/kg) and iron concentration (32.2 mg/kg). High heritability was found for days to heading (0.85), days to maturity (0.65), plant height (0.76), grain zinc concentration (0.81), and grain iron concentration (0.56), while moderate heritability was found for grain yield (0.33) and thousand grain weight (0.21). High genetic advance as a percentage of mean was found for grain iron concentration (50%), days to heading (24%), and grain yield (20%) (Table 2).

### Cluster Analysis

#### 6th HarvestPlus Yield Trial—2015–16

**Figure 1:**
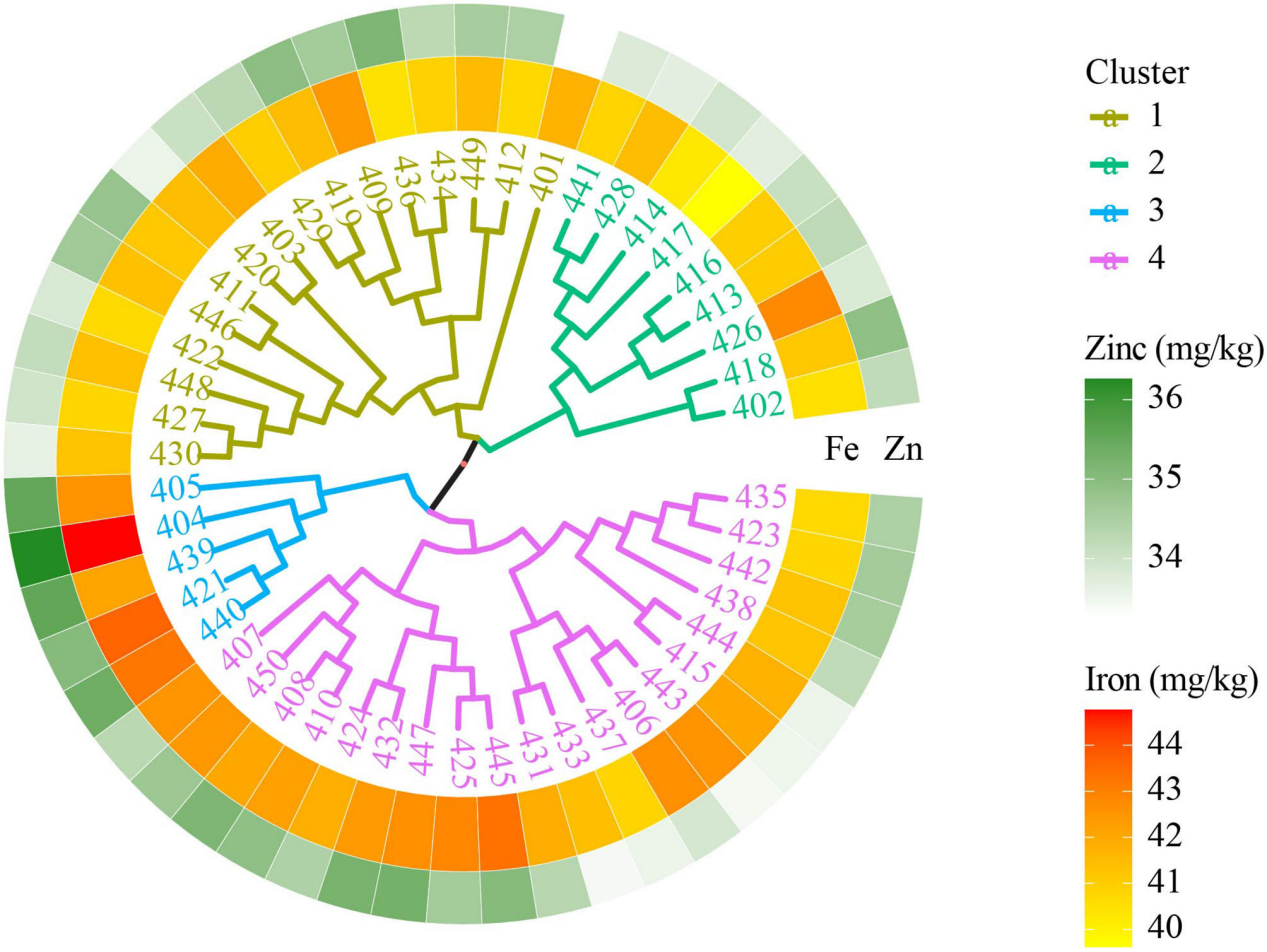
Clustering of biofortified bread wheat genotypes from the 6th HPYT based on BLUP values.

The 50 genotypes in the 6th HPYT were divided into four clusters based on days to heading, days to maturity, plant height, thousand grain weight, grain yield, and grain iron and zinc concentrations. The genotypes in cluster 4 were found to have high grain yield (3,346 kg/ha) with the heaviest thousand grain weight (49.9 gm), taller stature (91 cm), medium in grain iron (42.0 mg/kg), and zinc (34.4 mg/kg) concentration. However, the genotypes of cluster 3 had the lowest grain yield (3,252 kg/ha), and early maturing (152 days) but were rich in grain iron (43.3 mg/kg) and zinc concentrations (35.5 mg/kg). The genotypes in cluster 2 had the lowest grain zinc (34.0 mg/kg) and iron (41.0 mg/kg) concentrations (Table 3, Figure 1).

**Table 3:** Cluster mean of the 6th HarvestPlus Yield Trial (2015–16), 7th HarvestPlus Yield Trial (2016–17), 8th HarvestPlus Yield Trial (2017–18), and 9th HarvestPlus Yield Trial (2018–19).

**Cluster**	**No. of Genotypes**	**DTH**	**DTM**	**Pht**	**Tillers**	**SpkL**	**GNS**	**GWS**	**TGW**	**GY**	**Grain** **Fe**	**Grain** **Zn**	**Genotypes**
**6**^**th**^ **HPYT**													
1	16	112	167	88	X	X	X	X	46.5	3,312	41.3	34.3	401, 403, 409, 411, 412, 419, 420, 422, 427, 429, 430, 434, 436, 446, 448, 449
2	9	109	164	89	X	X	X	X	44.9	3,302	41	34	402, 413, 414, 416, 417, 418, 426, 428, 441
3	5	110	163	88	X	X	X	X	46.9	3,252	43.3	35.5	404, 405, 421, 439, 440
4	20	110	165	91	X	X	X	X	49.9	3,346	42	34.3	406, 407, 408, 410, 415, 423, 424, 425, 431, 432, 433, 435, 437, 438, 442, 443, 444, 445, 447, 450
**7**^**th**^ **HPYT**													
1	16	103	150	90	272	X	53	2.73	49.3	3,825	44.06	33.6	401, 402, 403, 405, 409, 414, 421, 428, 429, 430, 434, 437, 439, 443, 448, 450
2	1	100	144	92	270	X	46	2.51	52.7	3,495	47.46	38.1	404
3	17	102	149	91	253	X	51	2.77	52.9	3,709	44.26	33.7	406, 408, 413, 415, 416, 418, 419, 420, 423, 425, 427, 432, 433, 435, 438, 441, 442
4	8	105	151	92	255	X	54	2.96	54	3,768	44.17	33.9	407, 417, 422, 426, 436, 440, 444, 449
5	8	103	149	91	263	X	51	2.74	52.2	3,699	44.99	34.8	410, 411, 412, 424, 431, 445, 446, 447
**8**^**th**^ **HPYT**													
1	18	108	151	81	195	9.3	53	X	46.6	3,107	39.43	29.2	401, 403, 411, 425, 428, 432, 433, 434, 436, 437, 438, 439, 440, 441, 442, 444, 446, 447
2	6	103	144	86	200	9.3	53	X	49.5	3,288	41.35	30.1	402, 404, 406, 420, 435, 443
3	23	106	149	84	200	9.5	53	X	49.1	3,414	39.7	29.2	405, 407, 408, 409, 410, 412, 413, 414, 415, 416, 418, 419, 422, 423, 424, 426, 427, 429, 430, 431, 445, 448, 449
4	1	108	152	82	193	9.7	53	X	46.4	2,829	49.17	31.8	417
5	2	105	148	94	193	9.4	53	X	45.2	3,278	39.25	28.5	421, 450
**9**^**th**^ **HPYT**													
1	9	102	145	84	365	X	56	2.52	43.6	4,547	32	29.1	401, 402, 403, 420, 425, 426, 431, 436, 437
2	1	93	139	88	365	X	48	2.3	43.8	4,460	37	33.5	404
3	12	99	143	88	362	X	55	2.52	43.9	4,655	33	30	405, 421, 422, 427, 430, 432, 433, 440, 442, 447, 449, 450
4	23	101	144	87	355	X	57	2.58	43.9	4,596	33	30.3	406, 407, 408, 409, 412, 413, 414, 415, 416, 417, 418, 419, 424, 429, 434, 435, 438, 439, 441, 443, 444, 445, 446
5	5	99	143	87	362	X	54	2.48	43.8	4,564	33	30.7	410, 411, 423, 428, 448

*DTH, days to heading; DTM, days to maturity; Pht, plant height; SpkL, spike length; GNS, grain number per spike; GWS, grain weight per spike; TGW, thousand grain weight; GY, grain yield; GrainFe, grain iron concentration; GrainZn, grain zinc concentration*.

#### 7th HarvestPlus Yield Trial—2016–17

**Figure 2:**
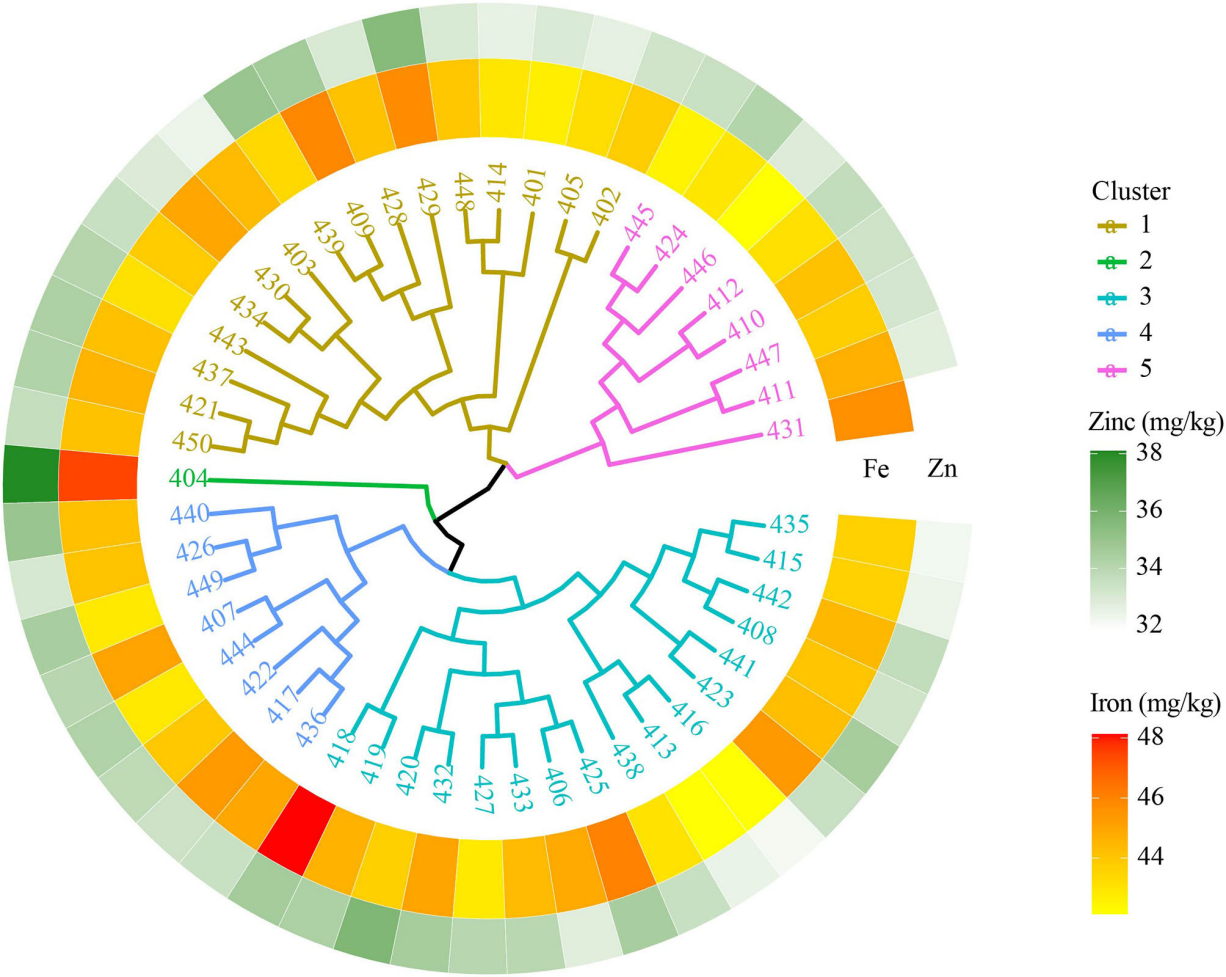
Clustering of biofortified bread wheat genotypes from the 7th HPYT based on BLUP values.

The 7th HPYT genotypes were divided into five clusters based on days to heading, days to maturity, plant height, the number of tillers per m^2^, grain numbers per spike, grain weight per spike, thousand grain weight, grain yield, and grain iron and zinc concentrations. Genotype 404 in cluster 2 had the highest grain iron (47.46 mg/kg) and zinc (38.1 mg/kg) concentrations but the lowest grain yield (3,495 kg/ha), and earlier maturity (144 days) than the other clusters. The 16 genotypes of cluster 1 had the highest grain yield (3,825 kg/ha) and the highest number of tillers per square meter (272). Cluster 4 shows the highest number of grains (54) and its weight per spike (2.96 g) and thousand grain weight (54.0 g) (Table 3, Figure 2).

#### 8th HarvestPlus Yield Trial–2017–18

**Figure 3:**
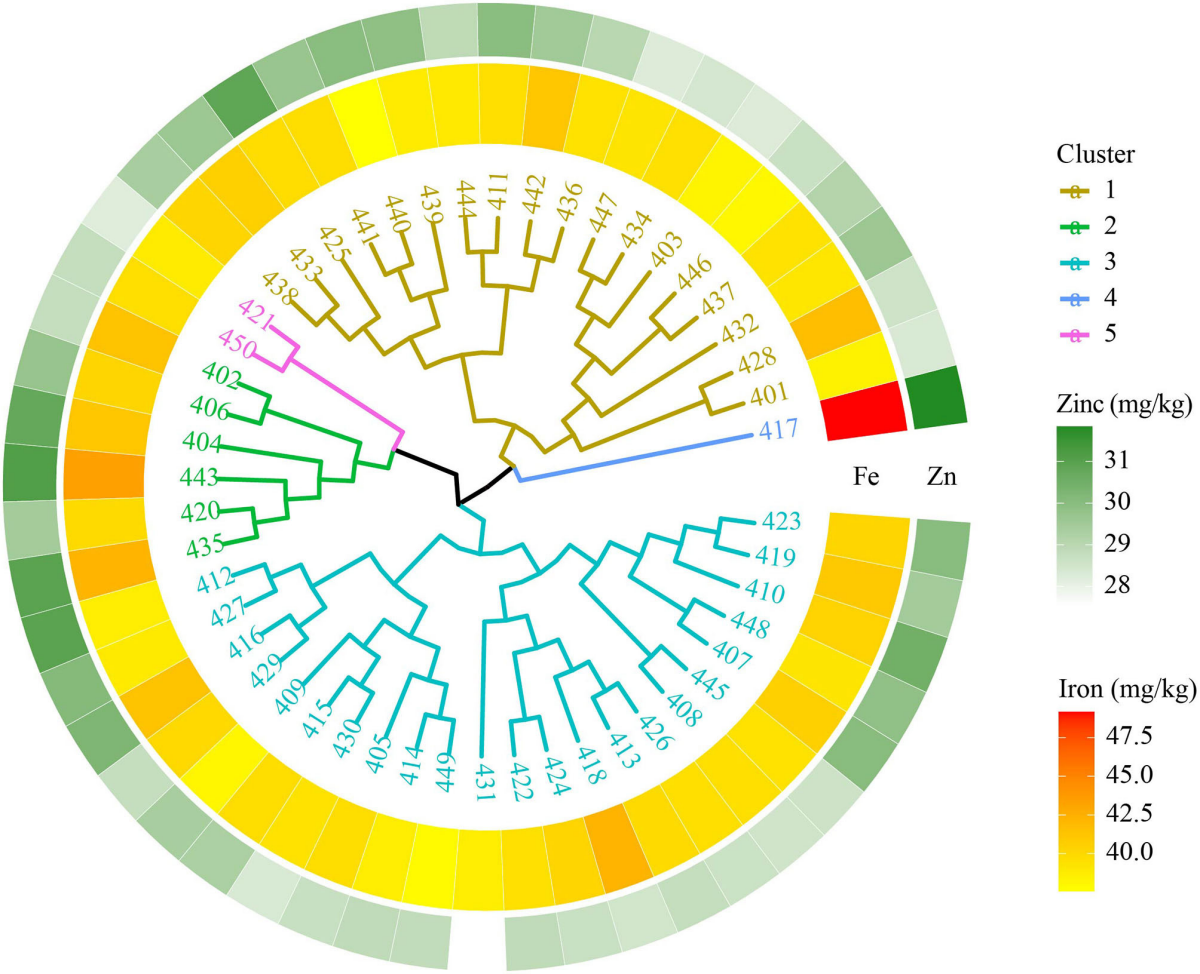
Clustering of biofortified bread wheat genotypes from the 8th HPYT based on BLUP values.

The 8th HPYT genotypes were divided into five clusters based on days to heading, days to maturity, plant height, spike length, number of tillers per m^2^, number of grains per spike, thousand grain weight, grain yield, and grain zinc and iron concentrations. Genotype 417 in cluster 4 had the highest grain zinc (31.83 mg/kg) and iron concentrations (49.17 mg/kg), but the grain yield (2,829 kg/ha) was low. The genotypes of cluster 3 had the highest grain yield (3,414 kg/ha) with a high number of tillers per m^2^ (200). The genotypes of cluster 2 had the shortest number of days to maturity (144 days) (Table 3, Figure 3).

#### 9th HarvestPlus Yield Trial—2018–19

**Figure 4:**
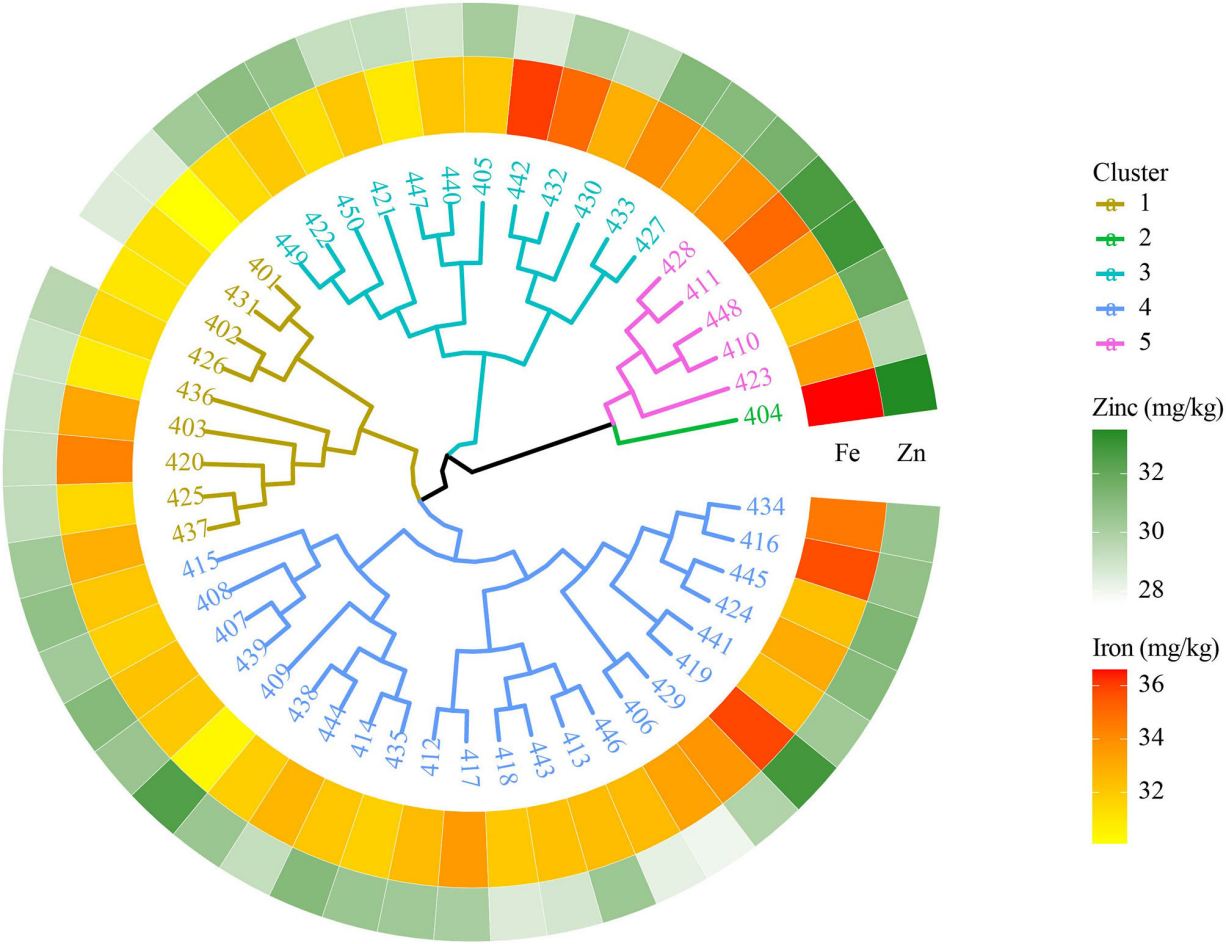
Clustering of biofortified bread wheat genotypes from the 9th HPYT based on BLUP values.

The 9th HPYT genotypes were divided into 5 clusters based on days to heading, days to maturity, plant height, number of tillers per m^2^, grain number per spike, grain weight per spike, thousand grain weight, grain yield, grain iron concentration, and grain zinc concentration. Among the clusters, cluster 2, which had only one genotype, entry 404, had the highest grain zinc (33.5 mg/kg), and iron concentrations (37 mg/kg) but the lowest grain yield (4,460 kg/ha). The genotypes of cluster 1 had the lowest grain iron (32 mg/kg) and zinc (29.1) concentrations. Grain yield (4,655 kg/ha) was found to be highest in cluster 3 (Table 3, Figure 4).

### Correlation Analysis

**Figure 5:**
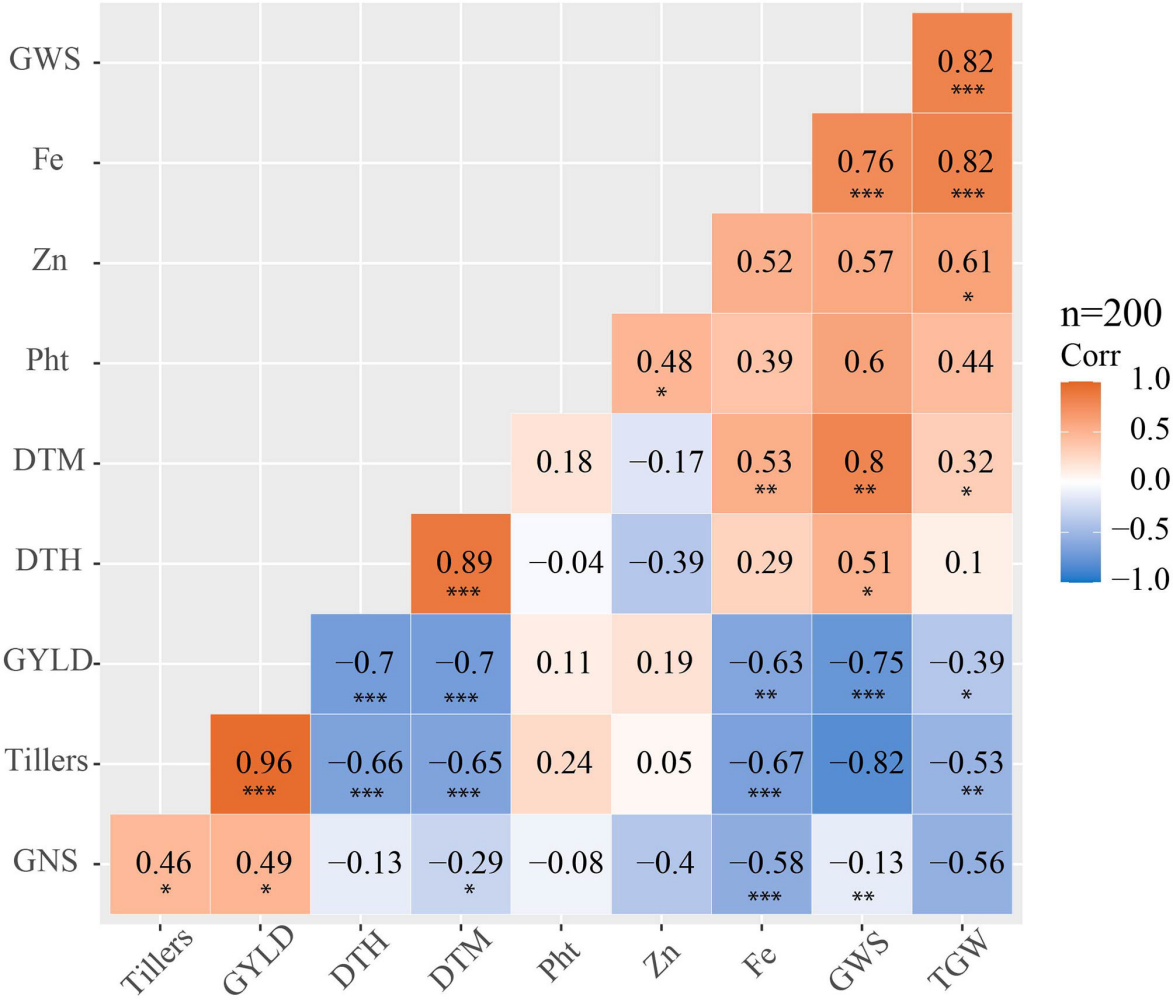
Phenotypic correlation matrix showing phenotypic correlation coefficients among the studied traits for yield, yield-related traits and zinc, and iron concentration in the 6th, 7th, 8th and 9th HPYTs (DTH, days to heading; DTM, days to maturity; Pht, plant height; GWS, grain weight per spike; GNS, grain number per spike; GY, grain yield; Fe, grain iron concentration; Zn, grain zinc concentration); the significance levels are indicated below the correlation coefficient as *: *p* < 0.05, **: *p* < 0.01 and ***: *p* < 0.001.

The phenotypic correlation analysis of the 6th, 7th, 8th and 9th HPYTs presented in Figure 5 shows that grain yield had a highly positive correlation with the number of tillers per m^2^ (0.96) and moderately positive correlation with grain number per spike (0.49) and grain zinc concentration (0.19). However, the correlation of grain yield with days to heading (−0.7), days to maturity (−0.7), grain iron concentration (−0.63), and thousand grain weight (−0.39) was found to be negative. The grain zinc concentration was positively correlated with plant height (0.48), grain iron concentration (0.52), grain weight per spike (0.57), and thousand grain weight (0.61). Similarly, the grain iron concentration also had a highly positive correlation with thousand grain weight (0.82), grain weight per spike (0.76), grain zinc concentration (0.52), and days to maturity (0.53).

## Discussion

Multi-environment testing is a crucial part of wheat breeding programs to identify stable and high-yielding lines with better nutritional quality. Therefore, this study showed variable responses of a set of new biofortified wheat genotypes across contrasting environments in Nepal. This is the first proof-of-concept results from multi-environment testing of high Zn biofortified wheat lines in Nepal across diverse growing environments. This study was conducted in a fast-track mode where the high zinc wheat trials from CIMMYT, Mexico introduced into Nepal which were grown across multi-environment to identify stable and high yielding genotypes which combine high yield, high Zn and Fe, and disease resistance traits in the target environments of Nepal.

The multi-environment data showed moderate heritability of grain yield, grain iron concentration, and grain zinc concentration in the 8th HPYT and these traits have the ability to transmit to the next generation. Grain yield had low heritability in both years, while grain iron concentration had moderate heritability in the 6th HPYT and low heritability in the 7th HPYT. The difference in the result might be due to various factors including different genetic materials used and different environmental conditions. Heritability for grain zinc concentration was moderate in all 3 years. Badakhshan et al. (2013) reported high heritability of grain zinc concentration. Despite the moderate heritability for grain Zn, the genotype × environment interaction specifically environmental factors such as available soil Zn, pH, CaCO_3_, and soil organic matters plays a major role in terms of grain Zn accumulation.

Among the locations, the grain iron concentration in Doti was found to be higher than in the other locations. This might be because of the higher soil iron concentration in Doti than in the other locations. However, the grain iron and zinc concentrations were very low in Surkhet compared with the other locations, perhaps because of the low iron and zinc concentrations in the soil in Surkhet.

The highly positive correlation of the number of tillers per square meter with grain yield shows that grain yield could be increased by increasing the number of tillers. Indirect selection for higher-grain-yielding genotypes could be done based on the number of tillers per square meter trait. Similar results were reported by Sharma (1995); Okuyama et al. (2004), and Waqar-Ul-Haq et al. (2010), while Kashif and Khaliq (2004) reported a highly negative correlation between grain yield and fertile tillers.

The positive correlation between the grain iron and grain zinc concentrations shows that these two traits can be improved simultaneously in a breeding program. This finding is in line with the studies by Morgounov et al. (2007); Velu et al. (2012); Liu et al. (2014); Chatrath et al. (2018), and Ghimire et al. (2019).

The negative correlation of grain iron with grain yield shows that increasing grain yield will decrease the grain iron concentration. Liu et al. (2014) and Chatrath et al. (2018) also reported a negative correlation of grain yield with grain iron. This might be due to the dilution effect (Oury et al., 2006; Liu et al., 2014). The positive correlation between grain yield and grain zinc shows that an increase in grain yield increases grain zinc concentration; Chatrath et al. (2018) also reported a similar relationship, although Liu et al. (2014) had reported a negative correlation of grain yield with grain zinc concentration.

The grain iron and zinc concentrations were positively correlated with plant height. This shows that taller genotypes yield more grain iron and zinc than dwarf ones. This might be explained by taller genotypes possibly having a lower dilution effect. According to Velu et al. (2017), the increase in grain zinc and iron in taller genotypes might be because of their larger kernel size and lower yield levels. However, Morgounov et al. (2007) reported a significant negative correlation of grain iron and zinc concentrations with plant height. The nutritionally-biofortified CIMMYT derived wheat genotypes evaluated in this study contain high yield and increased grain Zn over the local checks. This study led to the identification and release of five biofortified wheat varieties in Nepal. The new varieties including Zinc Gahun 1, Zinc Gahun 2, Bheri-Ganga, Himganga, and Khumal-Shakti were direct selections from the CIMMYT derived HarvestPlus Yield Trials which were evaluated across environments in Nepal. These varieties are replacing old disease susceptible varieties in Nepal and the fast-track seed multiplication and dissemination is underway to reach more farmers in the coming years.

## Conclusion

Large variations were found among the genotypes for grain yield, grain iron, and zinc among high zinc elite lines evaluated across environments in Nepal during the 4 years of testing, showing the remarkable potential for improving grain yields and grain iron and zinc concentrations. Spatial variability affected grain yields as well as grain iron and grain zinc concentrations. However, the multi-environment testing identified stable and promising genotypes suitable for diverse environments of Nepal ranging from hilly mountains to terai regions. The cluster analysis identified promising genotypes with high yields along with high grain zinc and iron concentrations which can be used in further breeding programs. Grain iron and zinc concentrations can be improved simultaneously during wheat breeding for better nutritional quality. Indirect selection for higher yield can be done based on a higher number of tillers per m^2^.

## Data Availability Statement

The datasets presented in this study can be found in online repositories. The names of the repository/repositories and accession number(s) can be found in the article/Supplementary Material.

## Author Contributions

DT thapa-field research, data analysis, and conceptualization of manuscript write up. MSu field research, data analysis, and manuscript write up. RY, BJ, BA, KS, PM, KP, SGu, SGh, NG, and NA field research and data generation at different research stations of Nepal agricultural research council. MSa data analysis and manuscript write up. VM grain zinc and iron analysis. AJ, RS, and VG providing germplasm for research and review and final manuscript edit. All authors contributed to the article and approved the submitted version.

## Funding

This research was supported by a grant from HarvestPlus Challenge Program, the Bill and Melinda Gates Foundation [INV-003012] and co-funded by the Foreign, Commonwealth and Development Office (FCDO) of the UK Government to CIMMYT.

## Conflict of Interest

The authors declare that the research was conducted in the absence of any commercial or financial relationships that could be construed as a potential conflict of interest.

## Publisher's Note

All claims expressed in this article are solely those of the authors and do not necessarily represent those of their affiliated organizations, or those of the publisher, the editors and the reviewers. Any product that may be evaluated in this article, or claim that may be made by its manufacturer, is not guaranteed or endorsed by the publisher.
