# Supplementary material for: Variation in Grain Zinc and Iron Concentrations, Grain Yield and Associated Traits of Biofortified Bread Wheat Genotypes in Nepal
Source: Front Plant Sci. 2022 Jun 13;13:881965. doi: 10.3389/fpls.2022.881965 (PMC9249123; doi:10.3389/fpls.2022.881965)
Supplement: Supplementary file 1 [file Data_Sheet_1.docx]

Supplementary Material

Supplementary Table 1 | Genotype details for 6^th^ HarvestPlus Yield Trial

| **EN** | **Cross Name** | **Selection History** |
| --- | --- | --- |
| 401 | WK1204 (=SW89.3064/STAR) (LOCAL CHECK) | CMBW91Y01627S-13Y-010M-010Y-010M-3Y-0M |
| 402 | BAJ #1 | CGSS01Y00134S-099Y-099M-099M-13Y-0B |
| 403 | KACHU #1 | CMSS97M03912T-040Y-020Y-030M-020Y-040M-4Y-2M-0Y |
| 404 | CROC_1/AE.SQUARROSA(210)//INQALAB 91*2/KUKUNA/3/PBW343*2/KUKUNA | CMSA06M00195T-099Y-099Y-9M-0Y-7B-0Y |
| 405 | WHEAR/KIRITATI/3/C80.1/3*BATAVIA//2*WBLL1/4/CMH75A.66/SERI | CMSS09Y00599S-099Y-17M-0WGY-8B-0Y |
| 406 | DANPHE #1*2/SOLALA/3/TACUPETO F2001/BRAMBLING*2//KACHU | CMSS11Y00458S-099Y-099M-3WGY-0B |
| 407 | DANPHE #1*2/SOLALA/3/TACUPETO F2001/BRAMBLING*2//KACHU | CMSS11Y00458S-099Y-099M-7WGY-0B |
| 408 | DANPHE #1*2/3/T.DICOCCON PI94625/AE.SQUARROSA (372)//SHA4/CHIL/6/WBLL1/3/STAR//KAUZ/STAR/4/BAV92/RAYON/5/TRAP#1/BOW/3/VEE/PJN//2*TUI/4/BAV92/RAYON | CMSS11Y00460S-099Y-099M-16WGY-0B |
| 409 | DANPHE #1*2/3/T.DICOCCON PI94625/AE.SQUARROSA (372)//SHA4/CHIL/6/WBLL1/3/STAR//KAUZ/STAR/4/BAV92/RAYON/5/TRAP#1/BOW/3/VEE/PJN//2*TUI/4/BAV92/RAYON | CMSS11Y00460S-099Y-099M-22WGY-0B |
| 410 | DANPHE #1*2/3/T.DICOCCON PI94625/AE.SQUARROSA (372)//SHA4/CHIL/6/WBLL1/3/STAR//KAUZ/STAR/4/BAV92/RAYON/5/TRAP#1/BOW/3/VEE/PJN//2*TUI/4/BAV92/RAYON | CMSS11Y00460S-099Y-099M-23WGY-0B |
| 411 | DANPHE #1*2/3/T.DICOCCON PI94625/AE.SQUARROSA (372)//SHA4/CHIL/6/WBLL1/3/STAR//KAUZ/STAR/4/BAV92/RAYON/5/TRAP#1/BOW/3/VEE/PJN//2*TUI/4/BAV92/RAYON | CMSS11Y00460S-099Y-099M-28WGY-0B |
| 412 | VILLA JUAREZ F2009/SOLALA//WBLL1*2/BRAMBLING/5/SSERI1/CHIBIA/4/BAV92//IRENA/KAUZ/3/HUITES | CMSS11Y00491S-099Y-099M-2WGY-0B |
| 413 | KVZ/PPR47.89C//FRANCOLIN #1/3/2*PAURAQ/4/PBW343*2/KUKUNA*2//FRTL/PIFED | CMSS11Y00499S-099Y-099M-1WGY-0B |
| 414 | KVZ/PPR47.89C//FRANCOLIN #1/3/2*PAURAQ/4/PBW343*2/KUKUNA*2//FRTL/PIFED | CMSS11Y00499S-099Y-099M-17WGY-0B |
| 415 | KVZ/PPR47.89C//FRANCOLIN #1/3/2*PAURAQ/4/UP2338*2/KKTS*2//YANAC | CMSS11Y00500S-099Y-099M-3WGY-0B |
| 416 | KVZ/PPR47.89C//FRANCOLIN #1/3/2*PAURAQ/5/BAV92//IRENA/KAUZ/3/HUITES*2/4/MURGA | CMSS11Y00501S-099Y-099M-15WGY-0B |
| 417 | T.DICOCCON CI9309/AE.SQUARROSA (409)//2*PANDORA/3/UP2338*2/KKTS*2//YANAC | CMSS11Y00513S-099Y-099M-1WGY-0B |
| 418 | T.DICOCCON CI9309/AE.SQUARROSA (409)//2*PANDORA/3/UP2338*2/KKTS*2//YANAC | CMSS11Y00513S-099Y-099M-9WGY-0B |
| 419 | T.DICOCCON CI9309/AE.SQUARROSA (409)//2*PANDORA/5/WAXWING/3/BL 1496/MILAN//PI 610750/4/FRNCLN | CMSS11Y00515S-099Y-099M-4WGY-0B |
| 420 | CROC_1/AE.SQUARROSA (210)//WBLL1*2/BRAMBLING/3/VILLA JUAREZ F2009/5/BAV92//IRENA/KAUZ/3/HUITES*2/4/MURGA | CMSS11Y00519S-099Y-099M-10WGY-0B |
| 421 | REH/HARE//2*BCN/3/CROC_1/AE.SQUARROSA (213)//PGO/4/HUITES/5/T.DICOCCON PI94624/AE.SQUARROSA (409)//BCN/6/REH/HARE//2*BCN/3/CROC_1/AE.SQUARROSA (213)//PGO/4/HUITES/7/MUTUS/8/BAV92//IRENA/KAUZ/3/HUITES*2/4/MURGA | CMSS11Y00575S-099Y-099M-2WGY-0B |
| 422 | NAC/TH.AC//3*PVN/3/MIRLO/BUC/4/2*PASTOR/5/T.DICOCCON PI94624/AE.SQUARROSA (409)//BCN/6/WBLL4//BABAX.1B.1B*2/PRL/3/PASTOR/7/SUP152 | CMSS11Y00581S-099Y-099M-13WGY-0B |
| 423 | PICUS/3/KAUZ*2/BOW//KAUZ/4/KKTS/5/T.SPELTA PI348530/6/2*FRANCOLIN #1/7/2*WBLL1/KUKUNA//TACUPETO F2001/3/BAJ #1 | CMSS11Y01271T-099TOPM-099Y-099M-13WGY-0B |
| 424 | REH/HARE//2*BCN/3/CROC_1/AE.SQUARROSA (213)//PGO/4/HUITES/5/T.DICOCCON PI94624/AE.SQUARROSA (409)//BCN/6/REH/HARE//2*BCN/3/CROC_1/AE.SQUARROSA (213)//PGO/4/HUITES/7/MUTUS/8/PBW343*2/KUKUNA*2//FRTL/PIFED/9/KACHU #1/KIRITATI//KACHU | CMSS11Y01280T-099TOPM-099Y-099M-31WGY-0B |
| 425 | REH/HARE//2*BCN/3/CROC_1/AE.SQUARROSA (213)//PGO/4/HUITES/5/T.DICOCCON PI94624/AE.SQUARROSA (409)//BCN/6/REH/HARE//2*BCN/3/CROC_1/AE.SQUARROSA (213)//PGO/4/HUITES/7/MUTUS/8/2*UP2338*2/KKTS*2//YANAC | CMSS11Y01281T-099TOPM-099Y-099M-11WGY-0B |
| 426 | INQALAB 91*2/TUKURU//T.SPELTA PI348599/3/2*INQALAB 91*2/KUKUNA/4/2*KINGBIRD #1//INQALAB 91*2/TUKURU | CMSS11Y01289T-099TOPM-099Y-099M-2WGY-0B |
| 427 | INQALAB 91*2/TUKURU//T.SPELTA PI348599/3/2*INQALAB 91*2/KUKUNA/4/2*KINGBIRD #1//INQALAB 91*2/TUKURU | CMSS11Y01289T-099TOPM-099Y-099M-10WGY-0B |
| 428 | INQALAB 91*2/TUKURU//T.SPELTA PI348599/3/2*INQALAB 91*2/KUKUNA/4/PBW343*2/KUKUNA//TECUE #1/5/PBW343*2/KUKUNA//TECUE #1 | CMSS11Y01290T-099TOPM-099Y-099M-22WGY-0B |
| 429 | BL 1724*2/3/T.DICOCCON PI272533/AE.SQUARROSA (458)//CMH81A.1261/VEE#10/4/2*UP2338*2/KKTS*2//YANAC | CMSS11Y01293T-099TOPM-099Y-099M-5WGY-0B |
| 430 | KACHU*2/5/WBLL1*2/TUKURU/3/T.DICOCCON PI94624/AE.SQUARROSA (409)//BCN/4/WBLL1*2/TUKURU | CMSS10B00672T-099TOPY-099M-099Y-10M-0WGY |
| 431 | PBW343*2/KUKUNA//PBW343*2/KUKUNA/3/IWA 8600211//2*PBW343*2/KUKUNA/4/PBW343*2/KUKUNA//TECUE #1 | CMSS10B00684T-099TOPY-099M-099Y-25M-0WGY |
| 432 | PBW343*2/KUKUNA//PBW343*2/KUKUNA/3/IWA 8600211//2*PBW343*2/KUKUNA/4/PBW343*2/KUKUNA//TECUE #1 | CMSS10B00684T-099TOPY-099M-099Y-50M-0WGY |
| 433 | MELON//FILIN/MILAN/3/FILIN/5/CROC_1/AE.SQUARROSA (444)/3/T.DICOCCON PI94625/AE.SQUARROSA (372)//3*PASTOR/4/T.DICOCCON PI94625/AE.SQUARROSA (372)//3*PASTOR/6/ATTILA/3*BCN//BAV92/3/TILHI/5/BAV92/3/PRL/SARA//TSI/VEE#5/4/CROC_1/AE.SQUARROSA (224)//2*OPAT | CMSS10B00690T-099TOPY-099M-099Y-7M-0WGY |
| 434 | MELON//FILIN/MILAN/3/FILIN/5/CROC_1/AE.SQUARROSA (444)/3/T.DICOCCON PI94625/AE.SQUARROSA (372)//3*PASTOR/4/T.DICOCCON PI94625/AE.SQUARROSA (372)//3*PASTOR/6/ATTILA/3*BCN//BAV92/3/TILHI/5/BAV92/3/PRL/SARA//TSI/VEE#5/4/CROC_1/AE.SQUARROSA (224)//2*OPAT | CMSS10B00690T-099TOPY-099M-099Y-12M-0WGY |
| 435 | MELON//FILIN/MILAN/3/FILIN/5/CROC_1/AE.SQUARROSA (444)/3/T.DICOCCON PI94625/AE.SQUARROSA (372)//3*PASTOR/4/T.DICOCCON PI94625/AE.SQUARROSA (372)//3*PASTOR/6/ATTILA/3*BCN//BAV92/3/TILHI/5/BAV92/3/PRL/SARA//TSI/VEE#5/4/CROC_1/AE.SQUARROSA (224)//2*OPAT | CMSS10B00690T-099TOPY-099M-099Y-20M-0WGY |
| 436 | MELON//FILIN/MILAN/3/FILIN/5/CROC_1/AE.SQUARROSA (444)/3/T.DICOCCON PI94625/AE.SQUARROSA (372)//3*PASTOR/4/T.DICOCCON PI94625/AE.SQUARROSA (372)//3*PASTOR/6/ATTILA/3*BCN//BAV92/3/TILHI/5/BAV92/3/PRL/SARA//TSI/VEE#5/4/CROC_1/AE.SQUARROSA (224)//2*OPAT | CMSS10B00690T-099TOPY-099M-099Y-34M-0WGY |
| 437 | FRET2/KUKUNA//FRET2/3/WHEAR/4/IWA 8600211//2*PBW343*2/KUKUNA/5/PBW343*2/KUKUNA//TECUE #1 | CMSS10B00699T-099TOPY-099M-099Y-1M-0WGY |
| 438 | FRET2/KUKUNA//FRET2/3/WHEAR/4/IWA 8600211//2*PBW343*2/KUKUNA/5/PBW343*2/KUKUNA//TECUE #1 | CMSS10B00699T-099TOPY-099M-099Y-2M-0WGY |
| 439 | THB/KEA//PF85487/3/DUCULA/4/WBLL1*2/TUKURU/5/IWA 8600211//2*PBW343*2/KUKUNA/6/TRCH/SRTU//KACHU | CMSS10B00708T-099TOPY-099M-099Y-6M-0WGY |
| 440 | TRCH/5/REH/HARE//2*BCN/3/CROC_1/AE.SQUARROSA (213)//PGO/4/HUITES/6/IWA 8600211//2*PBW343*2/KUKUNA/7/PBW343*2/KUKUNA*2//FRTL/PIFED | CMSS10B00722T-099TOPY-099M-099Y-19M-0WGY |
| 441 | TRCH/5/REH/HARE//2*BCN/3/CROC_1/AE.SQUARROSA (213)//PGO/4/HUITES/6/IWA 8600211//2*PBW343*2/KUKUNA/7/PBW343*2/KUKUNA*2//FRTL/PIFED | CMSS10B00722T-099TOPY-099M-099Y-42M-0WGY |
| 442 | TRCH/SRTU//KACHU*2/8/REH/HARE//2*BCN/3/CROC_1/AE.SQUARROSA (213)//PGO/4/HUITES/5/T.DICOCCON PI94624/AE.SQUARROSA (409)//BCN/6/REH/HARE//2*BCN/3/CROC_1/AE.SQUARROSA (213)//PGO/4/HUITES/7/MUTUS | CMSS10B00731T-099TOPY-099M-099Y-18M-0WGY |
| 443 | TRCH/SRTU//KACHU*2/8/REH/HARE//2*BCN/3/CROC_1/AE.SQUARROSA (213)//PGO/4/HUITES/5/T.DICOCCON PI94624/AE.SQUARROSA (409)//BCN/6/REH/HARE//2*BCN/3/CROC_1/AE.SQUARROSA (213)//PGO/4/HUITES/7/MUTUS | CMSS10B00731T-099TOPY-099M-099Y-19M-0WGY |
| 444 | TRCH/SRTU//KACHU*2/5/TOBA97/PASTOR/3/T.DICOCCON PI94624/AE.SQUARROSA (409)//BCN/4/BL 1496/MILAN//PI 610750 | CMSS10B00732T-099TOPY-099M-099Y-4M-0WGY |
| 445 | TRCH/SRTU//KACHU*2/5/TOBA97/PASTOR/3/T.DICOCCON PI94624/AE.SQUARROSA (409)//BCN/4/BL 1496/MILAN//PI 610750 | CMSS10B00732T-099TOPY-099M-099Y-13M-0WGY |
| 446 | SERI.1B//KAUZ/HEVO/3/AMAD*2/4/KIRITATI*2/6/BAV92//IRENA/KAUZ/3/HUITES/4/T.SPELTA PI348764/5/BAV92//IRENA/KAUZ/3/HUITES | CMSS10B00734T-099TOPY-099M-099Y-2M-0WGY |
| 447 | PSN/BOW//SERI/3/MILAN/4/ATTILA/5/KAUZ*2/CHEN//BCN/3/MILAN/6/WBLL1*2/4/SNI/TRAP#1/3/KAUZ*2/TRAP//KAUZ/7/IWA 8600211//2*PBW343*2/KUKUNA/8/PBW343*2/KUKUNA*2//FRTL/PIFED | CMSS10B00745T-099TOPY-099M-099Y-14M-0WGY |
| 448 | PICAFLOR #1/4/INQALAB 91*2/TUKURU//T.SPELTA PI348599/3/INQALAB 91*2/KUKUNA/5/KINGBIRD #1//INQALAB 91*2/TUKURU | CMSS10B00747T-099TOPY-099M-099Y-11M-0WGY |
| 449 | OASIS/SKAUZ//4*BCN/3/2*PASTOR/4/T.SPELTA PI348449/5/BACEU #1/6/WBLL1*2/CHAPIO/7/TACUPETO F2001/SAUAL//BLOUK #1 | CMSS10B00630S-099M-099Y-4M-0WGY |
| 450 | INQALAB 91*2/TUKURU//T.SPELTA PI348599/3/2*INQALAB 91*2/KUKUNA/4/KINGBIRD #1//INQALAB 91*2/TUKURU | CMSS10B00652S-099M-099Y-12M-0WGY |

Supplementary Table 2 | Genotype details for 7^th^ HarvestPlus Yield Trial

| **EN** | **Cross Name** | **Selection History** |
| --- | --- | --- |
| 401 | WK1204 (=SW89.3064/STAR) (LOCAL CHECK) | CMBW91Y01627S-13Y-010M-010Y-010M-3Y-0M |
| 402 | BAJ #1 | CGSS01Y00134S-099Y-099M-099M-13Y-0B |
| 403 | KACHU #1 | CMSS97M03912T-040Y-020Y-030M-020Y-040M-4Y-2M-0Y |
| 404 | CROC_1/AE.SQUARROSA (210)//INQALAB 91*2/KUKUNA/3/PBW343*2/KUKUNA | CMSA06M00195T-099Y-099Y-9M-0Y-7B-0Y |
| 405 | T.DICOCCON CI9309/AE.SQUARROSA (409)//MUTUS/3/2*MUTUS | CMSS08Y01129T-099M-099Y-3M-0Y-5M-0Y |
| 406 | DANPHE #1*2/3/T.DICOCCON PI94625/AE.SQUARROSA (372)//SHA4/CHIL/4/WBLL1*2/KURUKU//HEILO/5/WBLL1*2/KURUKU//HEILO | CMSS11B01191T-099TOPY-099M-099Y-3M-0WGY |
| 407 | DANPHE #1*2/3/T.DICOCCON PI94625/AE.SQUARROSA (372)//SHA4/CHIL/4/WBLL1*2/KURUKU//HEILO/5/WBLL1*2/KURUKU//HEILO | CMSS11B01191T-099TOPY-099M-099Y-30M-0WGY |
| 408 | DANPHE #1*2/3/T.DICOCCON PI94625/AE.SQUARROSA (372)//SHA4/CHIL/4/WBLL1*2/KURUKU//HEILO/5/WBLL1*2/KURUKU//HEILO | CMSS11B01191T-099TOPY-099M-099Y-31M-0WGY |
| 409 | CHONTE*2/SOLALA//2*BAJ #1 | CMSS11B01204T-099TOPY-099M-099Y-8M-0WGY |
| 410 | FRNCLN*2/7/CMH83.1020/HUITES/6/CMH79A.955/4/AGA/3/4*SN64/CNO67//INIA66/5/NAC/8/WBLL1*2/KURUKU//HEILO/9/WBLL1*2/KURUKU//HEILO | CMSS11B01210T-099TOPY-099M-099Y-9M-0WGY |
| 411 | FRNCLN*2/7/CMH83.1020/HUITES/6/CMH79A.955/4/AGA/3/4*SN64/CNO67//INIA66/5/NAC/8/WBLL1*2/KURUKU//HEILO/9/WBLL1*2/KURUKU//HEILO | CMSS11B01210T-099TOPY-099M-099Y-16M-0WGY |
| 412 | FRNCLN*2/7/CMH83.1020/HUITES/6/CMH79A.955/4/AGA/3/4*SN64/CNO67//INIA66/5/NAC/8/WBLL1*2/KURUKU//HEILO/9/WBLL1*2/KURUKU//HEILO | CMSS11B01210T-099TOPY-099M-099Y-51M-0WGY |
| 413 | FRNCLN*2/7/CMH83.1020/HUITES/6/CMH79A.955/4/AGA/3/4*SN64/CNO67//INIA66/5/NAC/8/KIRITATI/4/2*BAV92//IRENA/KAUZ/3/HUITES/9/FRANCOLIN #1//WBLL1*2/BRAMBLING | CMSS11B01213T-099TOPY-099M-099Y-8M-0WGY |
| 414 | VILLA JUAREZ F2009/SOLALA//WBLL1*2/BRAMBLING/5/WAXWING/3/BL 1496/MILAN//PI 610750/4/FRNCLN/6/MUNAL/3/HUW234+LR34/PRINIA//PFAU/WEAVER | CMSS11B01216T-099TOPY-099M-099Y-19M-0WGY |
| 415 | KVZ/PPR47.89C//TACUPETO F2001*2/BRAMBLING/3/2*TACUPETO F2001*2/BRAMBLING/4/KACHU/5/KACHU #1/3/C80.1/3*BATAVIA//2*WBLL1/4/KACHU | CMSS11B01218T-099TOPY-099M-099Y-7M-0WGY |
| 416 | KVZ/PPR47.89C//FRANCOLIN #1/3/2*PAURAQ/4/PBW343*2/KUKUNA*2//FRTL/PIFED/5/MUNAL #1 | CMSS11B01222T-099TOPY-099M-099Y-29M-0WGY |
| 417 | HGO94.7.1.12/2*QUAIU #1//QUAIU #2/3/KINGBIRD #1//INQALAB 91*2/TUKURU/4/SUP152/BAJ #1 | CMSS11B01227T-099TOPY-099M-099Y-13M-0WGY |
| 418 | HGO94.7.1.12/2*QUAIU #1//QUAIU #2/5/KIRITATI/4/2*BAV92//IRENA/KAUZ/3/HUITES/6/MUCUY | CMSS11B01228T-099TOPY-099M-099Y-14M-0WGY |
| 419 | HGO94.7.1.12/2*QUAIU #1//QUAIU #2/5/KIRITATI/4/2*BAV92//IRENA/KAUZ/3/HUITES/6/MUCUY | CMSS11B01228T-099TOPY-099M-099Y-20M-0WGY |
| 420 | CHIH95.2.6//WBLL1*2/KURUKU/3/WBLL1*2/KKTS/4/ND643/2*WBLL1/5/SAUAL/YANAC//SAUAL/6/WBLL1*2/BRAMBLING//VORB/FISCAL/3/BECARD | CMSS11B01230T-099TOPY-099M-099Y-19M-0WGY |
| 421 | CHIH95.2.6//WBLL1*2/KURUKU/3/WBLL1*2/KKTS/4/ND643/2*WBLL1/5/TACUPETO F2001/BRAMBLING*2//KACHU/6/KUTZ | CMSS11B01231T-099TOPY-099M-099Y-12M-0WGY |
| 422 | T.DICOCCON CI9309/AE.SQUARROSA (409)//2*PANDORA/3/KINGBIRD #1//INQALAB 91*2/TUKURU/5/MUNAL/3/KIRITATI//PRL/2*PASTOR/4/MUNAL | CMSS11B01236T-099TOPY-099M-099Y-31M-0WGY |
| 423 | T.DICOCCON CI9309/AE.SQUARROSA (409)//2*PANDORA/5/WAXWING/3/BL 1496/MILAN//PI 610750/4/FRNCLN/6/KACHU/BECARD//WBLL1*2/BRAMBLING | CMSS11B01237T-099TOPY-099M-099Y-3M-0WGY |
| 424 | HGO94.7.1.12/2*QUAIU #1//WAXBI/5/WBLL1*2/4/BABAX/LR42//BABAX/3/BABAX/LR42//BABAX | CMSS11B01246T-099TOPY-099M-099Y-23M-0WGY |
| 425 | COAH90.26.31/4/2*BL2064//SW89-5124*2/FASAN/3/TILHI/5/UP2338*2/KKTS*2//YANAC/6/MUTUS/AKURI | CMSS11B01249T-099TOPY-099M-099Y-19M-0WGY |
| 426 | COAH90.26.31/4/2*BL2064//SW89-5124*2/FASAN/3/TILHI/5/UP2338*2/KKTS*2//YANAC/6/MUTUS/AKURI | CMSS11B01249T-099TOPY-099M-099Y-32M-0WGY |
| 427 | CROC_1/AE.SQUARROSA (210)//INQALAB 91*2/KUKUNA/3/PBW343*2/KUKUNA/5/SAUAL/3/C80.1/3*BATAVIA//2*WBLL1/4/SITE/MO//PASTOR/3/TILHI/6/SAUAL #1/KACHU | CMSS11B01270T-099TOPY-099M-099Y-11M-0WGY |
| 428 | UC1113-GPCB1/3/TACUPETO F2001/BRAMBLING*2//KACHU/4/TACUPETO F2001/BRAMBLING//KACHU | CMSS11B01295T-099TOPY-099M-099Y-10M-0WGY |
| 429 | CHONTE*2/SOLALA/5/GARZA/BOY//AE.SQUARROSA (467)/3/T.DICOCCON PI94625/AE.SQUARROSA (372)//3*PASTOR/4/T.DICOCCON PI94625/AE.SQUARROSA (372)//3*PASTOR/6/ATTILA*2/PBW65//PIHA/3/ATTILA/2*PASTOR | CMSS11B01302T-099TOPY-099M-099Y-8M-0WGY |
| 430 | QUAIU #1/3/T.DICOCCON PI94625/AE.SQUARROSA (372)//3*PASTOR/4/QUAIU #2/5/BORL14 | CMSS11B01045S-099M-099Y-1M-0WGY |
| 431 | DANPHE #1*2/SOLALA/3/ATTILA*2/PBW65//MURGA | CMSS11B01057S-099M-099Y-17M-0WGY |
| 432 | VILLA JUAREZ F2009/SOLALA//WBLL1*2/BRAMBLING/4/COAH90.26.31//KIRITATI/WBLL1/3/KIRITATI/2*WBLL1 | CMSS11B01079S-099M-099Y-16M-0WGY |
| 433 | VILLA JUAREZ F2009/SOLALA//WBLL1*2/BRAMBLING/3/PBW343*2/KUKUNA*2//FRTL/PIFED | CMSS11B01081S-099M-099Y-11M-0WGY |
| 434 | VILLA JUAREZ F2009/SOLALA//WBLL1*2/BRAMBLING/3/PBW343*2/KUKUNA*2//FRTL/PIFED | CMSS11B01081S-099M-099Y-15M-0WGY |
| 435 | T.DICOCCON CI9309/AE.SQUARROSA (409)//MUTUS/3/2*MUTUS/5/T.DICOCCON PI94624/AE.SQUARROSA (409)//BCN/3/WAXWING/4/2*FRNCLN | CMSS11B01083S-099M-099Y-21M-0WGY |
| 436 | T.DICOCCON CI9309/AE.SQUARROSA (409)//MUTUS/3/2*MUTUS/4/FRET2/TUKURU//FRET2*2/3/T.SPELTA PI348530 | CMSS11B01084S-099M-099Y-6M-0WGY |
| 437 | T.DICOCCON CI9309/AE.SQUARROSA (409)//MUTUS/3/2*MUTUS/4/FRET2/TUKURU//FRET2*2/3/T.SPELTA PI348530 | CMSS11B01084S-099M-099Y-42M-0WGY |
| 438 | T.DICOCCON CI9309/AE.SQUARROSA (409)//MUTUS/3/2*MUTUS/5/PFAU/WEAVER*2/4/BOW/NKT//CBRD/3/CBRD | CMSS11B01087S-099M-099Y-21M-0WGY |
| 439 | T.DICOCCON PI94624/AE.SQUARROSA (409)//BCN/3/WAXWING/4/2*FRNCLN/5/VILLA JUAREZ F2009/3/T.DICOCCON PI94625/AE.SQUARROSA (372)//3*PASTOR/4/WBLL1*2/BRAMBLING | CMSS11B01090S-099M-099Y-1M-0WGY |
| 440 | CHIH95.2.6/4/BABAX/LR42//BABAX*2/3/SHAMA/5/2*BABAX/LR42//BABAX*2/3/TUKURU/6/KFA/2*KACHU | CMSS11B01099S-099M-099Y-5M-0WGY |
| 441 | HGO94.7.1.12/2*QUAIU #1/3/VILLA JUAREZ F2009/SOLALA//WBLL1*2/BRAMBLING | CMSS11B01126S-099M-099Y-8M-0WGY |
| 442 | HGO94.7.1.12//WBLL1*2/KUKUNA/3/WBLL1*2/KURUKU/4/PBW343*2/KUKUNA*2//FRTL/PIFED | CMSS11B01134S-099M-099Y-9M-0WGY |
| 443 | COAH90.26.31//KIRITATI/WBLL1/3/KIRITATI/2*WBLL1/7/OASIS/SKAUZ//4*BCN/3/2*PASTOR/4/T.SPELTA PI348449/5/BACEU #1/6/WBLL1*2/CHAPIO | CMSS11B01145S-099M-099Y-19M-0WGY |
| 444 | VILLA JUAREZ F2009/3/T.DICOCCON PI94625/AE.SQUARROSA (372)//3*PASTOR/4/WBLL1*2/BRAMBLING/5/QUAIU #1/3/T.DICOCCON PI94625/AE.SQUARROSA (372)//3*PASTOR/4/QUAIU #2 | CMSS11B01149S-099M-099Y-4M-0WGY |
| 445 | VILLA JUAREZ F2009/3/T.DICOCCON PI94625/AE.SQUARROSA (372)//3*PASTOR/4/WBLL1*2/BRAMBLING/5/QUAIU #1/3/T.DICOCCON PI94625/AE.SQUARROSA (372)//3*PASTOR/4/QUAIU #2 | CMSS11B01149S-099M-099Y-15M-0WGY |
| 446 | VILLA JUAREZ F2009/3/T.DICOCCON PI94625/AE.SQUARROSA (372)//3*PASTOR/4/WBLL1*2/BRAMBLING/5/QUAIU #1/3/T.DICOCCON PI94625/AE.SQUARROSA (372)//3*PASTOR/4/QUAIU #2 | CMSS11B01149S-099M-099Y-33M-0WGY |
| 447 | VILLA JUAREZ F2009/3/T.DICOCCON PI94625/AE.SQUARROSA (372)//3*PASTOR/4/WBLL1*2/BRAMBLING/7/OASIS/SKAUZ//4*BCN/3/2*PASTOR/4/T.SPELTA PI348449/5/BACEU #1/6/WBLL1*2/CHAPIO | CMSS11B01151S-099M-099Y-21M-0WGY |
| 448 | VILLA JUAREZ F2009/3/T.DICOCCON PI94625/AE.SQUARROSA (372)//3*PASTOR/4/WBLL1*2/BRAMBLING/5/BAJ #1/AKURI | CMSS11B01152S-099M-099Y-6M-0WGY |
| 449 | VILLA JUAREZ F2009/3/T.DICOCCON PI94625/AE.SQUARROSA (372)//3*PASTOR/4/WBLL1*2/BRAMBLING/5/ATTILA*2/PBW65//MUU #1/3/FRANCOLIN #1 | CMSS11B01153S-099M-099Y-14M-0WGY |
| 450 | 68.111/RGB-U//WARD/3/AE.SQUARROSA (321)/4/INQALAB 91*2/KUKUNA/5/PBW343*2/KUKUNA/6/MUCUY | CMSS11B01182S-099M-099Y-8M-0WGY |

Supplementary Table 3 | Genotype details for 8^th^ HarvestPlus Yield Trial

| **EN** | **Cross Name** | **Selection History** |
| --- | --- | --- |
| 401 | WK1204 (=SW89.3064/STAR) (LOCAL CHECK) | CMBW91Y01627S-13Y-010M-010Y-010M-3Y-0M |
| 402 | BAJ #1 | CGSS01Y00134S-099Y-099M-099M-13Y-0B |
| 403 | KACHU #1 | CMSS97M03912T-040Y-020Y-030M-020Y-040M-4Y-2M-0Y |
| 404 | ZINCSHAKTHI | CMSA06M00195T-099Y-099Y-9M-0Y-7B-0Y |
| 405 | MAYIL | CMSS08Y01129T-099M-099Y-3M-0Y-5M-0Y |
| 406 | DANPHE #1*2/3/T.DICOCCON PI94625/AE.SQUARROSA (372)//SHA4/CHIL/4/BOKOTA | CMSS12B01158S-099M-099Y-8M-0WGY |
| 407 | QUAIU #1/3/T.DICOCCON PI94625/AE.SQUARROSA (372)//3*PASTOR/4/QUAIU #2/5/VILLA JUAREZ F2009/3/T.DICOCCON PI94625/AE.SQUARROSA (372)//3*PASTOR/4/WBLL1*2/BRAMBLING | CMSS12B01180S-099M-099Y-2M-0WGY |
| 408 | QUAIU #1/3/T.DICOCCON PI94625/AE.SQUARROSA (372)//3*PASTOR/4/QUAIU #2/5/VILLA JUAREZ F2009/3/T.DICOCCON PI94625/AE.SQUARROSA (372)//3*PASTOR/4/WBLL1*2/BRAMBLING | CMSS12B01180S-099M-099Y-32M-0WGY |
| 409 | WHEAR/KUKUNA/3/C80.1/3*BATAVIA//2*WBLL1/4/T.DICOCCON PI94625/AE.SQUARROSA (372)//3*PASTOR/5/WHEAR/KUKUNA/3/C80.1/3*BATAVIA//2*WBLL1/6/QUAIU #1/SOLALA//QUAIU #2 | CMSS12B01199S-099M-099Y-13M-0WGY |
| 410 | MANKU/ZINCOL | CMSS12B01216S-099M-099Y-6M-0WGY |
| 411 | MANKU/ZINCOL | CMSS12B01216S-099M-099Y-35M-0WGY |
| 412 | KOKILA/BOKOTA | CMSS12B01232S-099M-099Y-12M-0WGY |
| 413 | ZINCOL/VALI | CMSS12B01234S-099M-099Y-10M-0WGY |
| 414 | ZINCOL/VALI | CMSS12B01234S-099M-099Y-29M-0WGY |
| 415 | PAURAQ//RL6043/4*NAC/3/QUAIU #1/SOLALA//QUAIU #2 | CMSS12B01290S-099M-099Y-10M-0WGY |
| 416 | DANPHE #1*2/3/T.DICOCCON PI94625/AE.SQUARROSA (372)//SHA4/CHIL/4/SHAKTI/5/VALI | CMSS12B01359T-099TOPY-099M-099Y-32M-0WGY |
| 417 | VALI*2/6/WHEAR/KUKUNA/3/C80.1/3*BATAVIA//2*WBLL1/4/T.DICOCCON PI94625/AE.SQUARROSA (372)//SHA4/CHIL/5/WHEAR/KUKUNA/3/C80.1/3*BATAVIA//2*WBLL1 | CMSS12B01362T-099TOPY-099M-099Y-56M-0WGY |
| 418 | VALI/6/2*WHEAR/KUKUNA/3/C80.1/3*BATAVIA//2*WBLL1/4/T.DICOCCON PI94625/AE.SQUARROSA (372)//SHA4/CHIL/5/WHEAR/KUKUNA/3/C80.1/3*BATAVIA//2*WBLL1 | CMSS12B01363T-099TOPY-099M-099Y-20M-0WGY |
| 419 | VALI/MAYIL/6/WHEAR/KUKUNA/3/C80.1/3*BATAVIA//2*WBLL1/4/T.DICOCCON PI94625/AE.SQUARROSA (372)//SHA4/CHIL/5/WHEAR/KUKUNA/3/C80.1/3*BATAVIA//2*WBLL1 | CMSS12B01366T-099TOPY-099M-099Y-17M-0WGY |
| 420 | VALI/MAYIL/6/WHEAR/KUKUNA/3/C80.1/3*BATAVIA//2*WBLL1/4/T.DICOCCON PI94625/AE.SQUARROSA (372)//SHA4/CHIL/5/WHEAR/KUKUNA/3/C80.1/3*BATAVIA//2*WBLL1 | CMSS12B01366T-099TOPY-099M-099Y-40M-0WGY |
| 421 | WHEAR/KUKUNA/3/C80.1/3*BATAVIA//2*WBLL1/4/T.DICOCCON PI94625/AE.SQUARROSA (372)//SHA4/CHIL/5/WHEAR/KUKUNA/3/C80.1/3*BATAVIA//2*WBLL1*2/6/ZINCOL | CMSS12B01368T-099TOPY-099M-099Y-22M-0WGY |
| 422 | COAH90.26.31//KIRITATI/WBLL1/3/KIRITATI/2*WBLL1/6/2*WHEAR/KUKUNA/3/C80.1/3*BATAVIA//2*WBLL1/4/T.DICOCCON PI94625/AE.SQUARROSA (372)//SHA4/CHIL/5/WHEAR/KUKUNA/3/C80.1/3*BATAVIA//2*WBLL1 | CMSS12B01374T-099TOPY-099M-099Y-52M-0WGY |
| 423 | QUAIU #1/3/T.DICOCCON PI94625/AE.SQUARROSA (372)//3*PASTOR/4/QUAIU #2/5/VALI/6/BECARD/QUAIU #1 | CMSS12B01377T-099TOPY-099M-099Y-8M-0WGY |
| 424 | QUAIU #1/3/T.DICOCCON PI94625/AE.SQUARROSA (372)//3*PASTOR/4/QUAIU #2/5/VALI/6/BECARD/QUAIU #1 | CMSS12B01377T-099TOPY-099M-099Y-10M-0WGY |
| 425 | MAYIL/ZINCOL//ITP40/AKURI | CMSS12B01380T-099TOPY-099M-099Y-40M-0WGY |
| 426 | HOLO/BORL14//VALI | CMSS12B01392T-099TOPY-099M-099Y-23M-0WGY |
| 427 | REH/HARE//2*BCN/3/CROC_1/AE.SQUARROSA (213)//PGO/4/HUITES/5/T.SPELTA PI348599/6/REH/HARE//2*BCN/3/CROC_1/AE.SQUARROSA (213)//PGO/4/HUITES/7/QUAIU/8/2*QUAIU #1/SOLALA//QUAIU #2 | CMSS12B01406T-099TOPY-099M-099Y-22M-0WGY |
| 428 | FRET2/TUKURU//FRET2*2/3/T.SPELTA PI348530/4/VALI/5/MUCUY | CMSS12B01408T-099TOPY-099M-099Y-39M-0WGY |
| 429 | KATERE/3/QUAIU #1/SOLALA//QUAIU #2/4/BECARD/QUAIU #1 | CMSS12B01418T-099TOPY-099M-099Y-21M-0WGY |
| 430 | KATERE/2*BORL14 | CMSS12B01419T-099TOPY-099M-099Y-16M-0WGY |
| 431 | KATERE/BORL14/3/WBLL1*2/KURUKU//SUP152 | CMSS12B01420T-099TOPY-099M-099Y-18M-0WGY |
| 432 | KATERE/BORL14/3/WBLL1*2/KURUKU//SUP152 | CMSS12B01420T-099TOPY-099M-099Y-30M-0WGY |
| 433 | CROC_1/AE.SQUARROSA (210)//PBW343*2/KUKUNA/3/PBW343*2/KUKUNA/4/VALI/5/MANKU | CMSS12B01424T-099TOPY-099M-099Y-24M-0WGY |
| 434 | SHAKTI/2*BORL14 | CMSS12B01430T-099TOPY-099M-099Y-27M-0WGY |
| 435 | VALI/MAYIL | CMSS12Y01314S-099Y-099M-099Y-6M-0WGY |
| 436 | WHEAR/KUKUNA/3/C80.1/3*BATAVIA//2*WBLL1/4/T.DICOCCON PI94625/AE.SQUARROSA (372)//SHA4/CHIL/5/WHEAR/KUKUNA/3/C80.1/3*BATAVIA//2*WBLL1/6/DANPHE #1*2/3/T.DICOCCON PI94625/AE.SQUARROSA (372)//SHA4/CHIL | CMSS12Y01317S-099Y-099M-099Y-12M-0WGY |
| 437 | WHEAR/KUKUNA/3/C80.1/3*BATAVIA//2*WBLL1/4/T.DICOCCON PI94625/AE.SQUARROSA (372)//SHA4/CHIL/5/WHEAR/KUKUNA/3/C80.1/3*BATAVIA//2*WBLL1/6/ZINCOL | CMSS12Y01319S-099Y-099M-099Y-24M-0WGY |
| 438 | MAYIL/ZINCOL | CMSS12Y01376S-099Y-099M-099Y-12M-0WGY |
| 439 | HOLO/VALI | CMSS12Y01405S-099Y-099M-099Y-27M-0WGY |
| 440 | VILLA JUAREZ F2009/3/T.DICOCCON PI94625/AE.SQUARROSA (372)//3*PASTOR/4/WBLL1*2/BRAMBLING/5/VALI | CMSS12Y01415S-099Y-099M-099Y-6M-0WGY |
| 441 | VILLA JUAREZ F2009/3/T.DICOCCON PI94625/AE.SQUARROSA (372)//3*PASTOR/4/WBLL1*2/BRAMBLING/5/VALI | CMSS12Y01415S-099Y-099M-099Y-12M-0WGY |
| 442 | ZINCOL/3/QUAIU #1/SOLALA//QUAIU #2 | CMSS12Y01432S-099Y-099M-099Y-15M-0WGY |
| 443 | REH/HARE//2*BCN/3/CROC_1/AE.SQUARROSA (213)//PGO/4/HUITES/5/T.SPELTA PI348599/6/REH/HARE//2*BCN/3/CROC_1/AE.SQUARROSA (213)//PGO/4/HUITES/7/QUAIU/8/KFA/2*KACHU | CMSS12Y01444S-099Y-099M-099Y-33M-0WGY |
| 444 | CROC_1/AE.SQUARROSA (210)//PBW343*2/KHVAKI/3/PBW343*2/KUKUNA/4/VALI | CMSS12Y01479S-099Y-099M-099Y-34M-0WGY |
| 445 | QUAIU #1/3/T.DICOCCON PI94625/AE.SQUARROSA (372)//3*PASTOR/4/QUAIU #2/5/CHONTE*2/3/T.DICOCCON PI94625/AE.SQUARROSA (372)//3*PASTOR/6/VALI | CMSS12Y01494T-099TOPM-099Y-099M-099Y-11M-0WGY |
| 446 | QUAIU #1/3/T.DICOCCON PI94625/AE.SQUARROSA (372)//3*PASTOR/4/QUAIU #2/5/CHONTE*2/3/T.DICOCCON PI94625/AE.SQUARROSA (372)//3*PASTOR/6/VALI | CMSS12Y01494T-099TOPM-099Y-099M-099Y-14M-0WGY |
| 447 | QUAIU #1/3/T.DICOCCON PI94625/AE.SQUARROSA (372)//3*PASTOR/4/QUAIU #2/5/CHONTE*2/3/T.DICOCCON PI94625/AE.SQUARROSA (372)//3*PASTOR/6/VALI | CMSS12Y01494T-099TOPM-099Y-099M-099Y-30M-0WGY |
| 448 | HGO94.7.1.12/2*QUAIU #1/6/CHIH95.2.6/4/BABAX/LR42//BABAX*2/3/SHAMA/5/2*BABAX/LR42//BABAX*2/3/TUKURU/7/SUP152 | CMSS12Y01554T-099TOPM-099Y-099M-099Y-36M-0WGY |
| 449 | VILLA JUAREZ F2009/3/T.DICOCCON PI94625/AE.SQUARROSA (372)//3*PASTOR/4/WBLL1*2/BRAMBLING/5/QUAIU #1/3/T.DICOCCON PI94625/AE.SQUARROSA (372)//3*PASTOR/4/QUAIU #2/6/QUAIU #1/SOLALA//QUAIU #2 | CMSS12Y01570T-099TOPM-099Y-099M-099Y-11M-0WGY |
| 450 | 68.111/RGB-U//WARD/3/AE.SQUARROSA (321)/4/INQALAB 91*2/KUKUNA/5/PBW343*2/KUKUNA/6/MUCUY/7/MAYIL | CMSS12Y01594T-099TOPM-099Y-099M-099Y-15M-0WGY |

Supplementary Table 4 | Genotype details for 9^th^ HarvestPlus Yield Trial

| EN | Cross Name | Selection History |
| --- | --- | --- |
| 401 | WK1204 (=SW89.3064/STAR) (LOCAL CHECK) | CMBW91Y01627S-13Y-010M-010Y-010M-3Y-0M |
| 402 | KACHU #1 | CMSS97M03912T-040Y-020Y-030M-020Y-040M-4Y-2M-0Y |
| 403 | MAYIL | CMSS08Y01129T-099M-099Y-3M-0Y-5M-0Y |
| 404 | ZINCSHAKTHI | CMSA06M00195T-099Y-099Y-9M-0Y-7B-0Y |
| 405 | DANPHE #1*2/SOLALA//BORL14 | CMSS13B00868S-099M-099Y-18M-0WGY |
| 406 | DANPHE #1*2/SOLALA//BORL14 | CMSS13B00868S-099M-099Y-25M-0WGY |
| 407 | VALI//KACHU/KIRITATI | CMSS13B00880S-099M-099Y-5M-0WGY |
| 408 | MANKU//MUTUS*2/TECUE #1 | CMSS13B00893S-099M-099Y-29M-0WGY |
| 409 | VILLA JUAREZ F2009/3/T.DICOCCON PI94625/AE.SQUARROSA (372)//3*PASTOR/4/WBLL1*2/BRAMBLING/5/WBLL1*2/BRAMBLING//QUAIU | CMSS13B00898S-099M-099Y-17M-0WGY |
| 410 | FRANCOLIN #1/3/IWA 8600211//2*PBW343*2/KUKUNA/7/TRAP#1/BOW/3/VEE/PJN//2*TUI/4/BAV92/RAYON/5/KACHU #1/6/TOBA97/PASTOR/3/T.DICOCCON PI94624/AE.SQUARROSA (409)//BCN/4/BL 1496/MILAN/3/CROC_1/AE.SQUARROSA (205)//KAUZ | CMSS13B00948S-099M-099Y-22M-0WGY |
| 411 | C80.1/3*BATAVIA//2*WBLL1/3/ATTILA/3*BCN*2//BAV92/4/WBLL1*2/KURUKU/5/IWA 8600211//2*PBW343*2/KUKUNA/7/TRAP#1/BOW/3/VEE/PJN//2*TUI/4/BAV92/RAYON/5/KACHU #1/6/TOBA97/PASTOR/3/T.DICOCCON PI94624/AE.SQUARROSA (409)//BCN/4/BL 1496/MILAN/3/CROC_1/AE.SQUARRO | CMSS13B00984S-099M-099Y-8M-0WGY |
| 412 | C80.1/3*BATAVIA//2*WBLL1/3/ATTILA/3*BCN*2//BAV92/4/WBLL1*2/KURUKU/5/IWA 8600211//2*PBW343*2/KUKUNA/6/MUCUY | CMSS13B00989S-099M-099Y-13M-0WGY |
| 413 | C80.1/3*BATAVIA//2*WBLL1/3/ATTILA/3*BCN*2//BAV92/4/WBLL1*2/KURUKU/5/IWA 8600211//2*PBW343*2/KUKUNA/6/MUCUY | CMSS13B00989S-099M-099Y-17M-0WGY |
| 414 | TRAP#1/BOW/3/VEE/PJN//2*TUI/4/BAV92/RAYON/5/KACHU #1/6/TOBA97/PASTOR/3/T.DICOCCON PI94624/AE.SQUARROSA (409)//BCN/4/BL 1496/MILAN/3/CROC_1/AE.SQUARROSA (205)//KAUZ/7/FRNCLN/3/ND643//2*PRL/2*PASTOR/4/FRANCOLIN #1 | CMSS13B01010S-099M-099Y-26M-0WGY |
| 415 | ROLF07*2/KIRITATI/3/IWA 8600211//2*PBW343*2/KUKUNA/4/MANKU | CMSS13B01018S-099M-099Y-22M-0WGY |
| 416 | SHAKTI/2*BORL14 | CMSS13B01796T-099TOPY-099M-099Y-6M-0WGY |
| 417 | SHAKTI/2*BORL14 | CMSS13B01796T-099TOPY-099M-099Y-16M-0WGY |
| 418 | SHAKTI/2*BORL14 | CMSS13B01796T-099TOPY-099M-099Y-35M-0WGY |
| 419 | SHAKTI/2*MUCUY | CMSS13B01802T-099TOPY-099M-099Y-17M-0WGY |
| 420 | SHAKTI/6/KAUZ//ALTAR 84/AOS/3/PASTOR/4/873.97/5/MUNAL #1/7/FRET2*2/SHAMA//KIRITATI/2*TRCH/3/BAJ #1 | CMSS13B01835T-099TOPY-099M-099Y-19M-0WGY |
| 421 | SHAKTI/7/SERI.1B*2/3/KAUZ*2/BOW//KAUZ/4/KRONSTAD F2004/5/MUNAL/6/MUNAL #1/8/MP4010/MUNAL #1 | CMSS13B01842T-099TOPY-099M-099Y-29M-0WGY |
| 422 | SHAKTI/5/WHEAR/KIRITATI/3/C80.1/3*BATAVIA//2*WBLL1*2/4/KIRITATI/2*TRCH/6/BECARD//KIRITATI/2*TRCH/3/BECARD | CMSS13B01843T-099TOPY-099M-099Y-12M-0WGY |
| 423 | KATERE/MUCUY/7/TRAP#1/BOW/3/VEE/PJN//2*TUI/4/BAV92/RAYON/5/KACHU #1/6/TOBA97/PASTOR/3/T.DICOCCON PI94624/AE.SQUARROSA (409)//BCN/4/BL 1496/MILAN/3/CROC_1/AE.SQUARROSA (205)//KAUZ | CMSS13B01852T-099TOPY-099M-099Y-4M-0WGY |
| 424 | KATERE/MUCUY/7/TRAP#1/BOW/3/VEE/PJN//2*TUI/4/BAV92/RAYON/5/KACHU #1/6/TOBA97/PASTOR/3/T.DICOCCON PI94624/AE.SQUARROSA (409)//BCN/4/BL 1496/MILAN/3/CROC_1/AE.SQUARROSA (205)//KAUZ | CMSS13B01852T-099TOPY-099M-099Y-22M-0WGY |
| 425 | KATERE//ONIX/KBIRD/6/C80.1/3*BATAVIA//2*WBLL1/3/ATTILA/3*BCN*2//BAV92/4/WBLL1*2/KURUKU/5/IWA 8600211//2*PBW343*2/KUKUNA | CMSS13B01854T-099TOPY-099M-099Y-8M-0WGY |
| 426 | ZINCOL//BECARD/QUAIU #1/7/INQALAB 91*2/TUKURU//WHEAR/6/BAV92//IRENA/KAUZ/3/HUITES/4/T.SPELTA PI348764/5/BAV92//IRENA/KAUZ/3/HUITES | CMSS13B01862T-099TOPY-099M-099Y-17M-0WGY |
| 427 | DANPHE #1*2/3/T.DICOCCON PI94625/AE.SQUARROSA (372)//SHA4/CHIL/4/PBW343*2/KUKUNA//PARUS/3/PBW343*2/KUKUNA/5/MAYIL | CMSS13B01878T-099TOPY-099M-099Y-8M-0WGY |
| 428 | HGO94.7.1.12//WBLL1*2/KUKUNA/3/WBLL1*2/KURUKU/4/PBW343*2/KUKUNA*2//FRTL/PIFED/6/C80.1/3*BATAVIA//2*WBLL1/3/ATTILA/3*BCN*2//BAV92/4/WBLL1*2/KURUKU/5/IWA 8600211//2*PBW343*2/KUKUNA | CMSS13B01895T-099TOPY-099M-099Y-3M-0WGY |
| 429 | VALI/3/MUTUS*2//ND643/2*WBLL1/6/C80.1/3*BATAVIA//2*WBLL1/3/ATTILA/3*BCN*2//BAV92/4/WBLL1*2/KURUKU/5/IWA 8600211//2*PBW343*2/KUKUNA | CMSS13B01911T-099TOPY-099M-099Y-5M-0WGY |
| 430 | WHEAR/KUKUNA/3/C80.1/3*BATAVIA//2*WBLL1/4/T.DICOCCON PI94625/AE.SQUARROSA (372)//SHA4/CHIL/5/WHEAR/KUKUNA/3/C80.1/3*BATAVIA//2*WBLL1/6/VILLA JUAREZ F2009/3/T.DICOCCON PI94625/AE.SQUARROSA (372)//3*PASTOR/4/WBLL1*2/BRAMBLING/7/TRAP#1/BOW/3/VEE/PJN//2* | CMSS13B01913T-099TOPY-099M-099Y-23M-0WGY |
| 431 | QUAIU #1/SOLALA//QUAIU #2/3/MANKU/4/KACHU #1/KIRITATI//KACHU | CMSS13B01918T-099TOPY-099M-099Y-5M-0WGY |
| 432 | KOKILA/3/MUTUS*2//ND643/2*WBLL1/8/PSN/BOW//SERI/3/MILAN/4/ATTILA/5/KAUZ*2/CHEN//BCN/3/MILAN/6/WBLL1*2/SHAMA/7/IWA 8600211//2*PBW343*2/KUKUNA | CMSS13B01939T-099TOPY-099M-099Y-16M-0WGY |
| 433 | KIRITATI/4/2*SERI.1B*2/3/KAUZ*2/BOW//KAUZ/5/CMH81.530/6/WHEAR/KIRITATI/3/C80.1/3*BATAVIA//2*WBLL1/4/CMH75A.66/SERI/7/VILLA JUAREZ F2009/3/T.DICOCCON PI94625/AE.SQUARROSA (372)//3*PASTOR/4/WBLL1*2/BRAMBLING | CMSS13B01941T-099TOPY-099M-099Y-4M-0WGY |
| 434 | WHEAR/KIRITATI/3/C80.1/3*BATAVIA//2*WBLL1/4/CMH75A.66/SERI/5/HUW234+LR34/PRINIA//PFAU/WEAVER/3/CMH83.30/6/KACHU/DANPHE | CMSS13B01946T-099TOPY-099M-099Y-1M-0WGY |
| 435 | DANPHE #1*2/3/T.DICOCCON PI94625/AE.SQUARROSA (372)//SHA4/CHIL/4/MANKU | CMSS13Y00823S-099Y-099M-099Y-36M-0WGY |
| 436 | WHEAR/KUKUNA/3/C80.1/3*BATAVIA//2*WBLL1/4/T.DICOCCON PI94625/AE.SQUARROSA (372)//SHA4/CHIL/5/WHEAR/KUKUNA/3/C80.1/3*BATAVIA//2*WBLL1/6/MUU/FRNCLN//FRANCOLIN #1 | CMSS13Y00867S-099Y-099M-099Y-13M-0WGY |
| 437 | MANKU/6/WHEAR/KUKUNA/3/C80.1/3*BATAVIA//2*WBLL1/5/PRL/2*PASTOR/4/CHOIX/STAR/3/HE1/3*CNO79//2*SERI | CMSS13Y00884S-099Y-099M-099Y-8M-0WGY |
| 438 | VILLA JUAREZ F2009/3/T.DICOCCON PI94625/AE.SQUARROSA (372)//3*PASTOR/4/WBLL1*2/BRAMBLING/5/WHEAR/KIRITATI/3/C80.1/3*BATAVIA//2*WBLL1*2/4/KIRITATI/2*TRCH | CMSS13Y00894S-099Y-099M-099Y-34M-0WGY |
| 439 | VALI/5/2*VILLA JUAREZ F2009/3/T.DICOCCON PI94625/AE.SQUARROSA (372)//3*PASTOR/4/WBLL1*2/BRAMBLING | CMSS13Y01564T-099TOPM-099Y-099M-099Y-18M-0WGY |
| 440 | QUAIU #1/3/T.DICOCCON PI94625/AE.SQUARROSA (372)//3*PASTOR/4/QUAIU #2*2/5/SUP152/BECARD | CMSS13Y01584T-099TOPM-099Y-099M-099Y-16M-0WGY |
| 441 | MAYIL/2*VALI | CMSS13Y01585T-099TOPM-099Y-099M-099Y-8M-0WGY |
| 442 | MAYIL/2*VALI | CMSS13Y01585T-099TOPM-099Y-099M-099Y-13M-0WGY |
| 443 | MAYIL*2//SUP152*2/TECUE #1 | CMSS13Y01587T-099TOPM-099Y-099M-099Y-54M-0WGY |
| 444 | VILLA JUAREZ F2009/3/T.DICOCCON PI94625/AE.SQUARROSA (372)//3*PASTOR/4/WBLL1*2/BRAMBLING*2/5/KVZ/PPR47.89C//TACUPETO F2001*2/BRAMBLING/3/2*TACUPETO F2001*2/BRAMBLING | CMSS13Y01615T-099TOPM-099Y-099M-099Y-55M-0WGY |
| 445 | KOKILA/2*VALI | CMSS13Y01619T-099TOPM-099Y-099M-099Y-7M-0WGY |
| 446 | SHAKTI/2*KUTZ | CMSS13Y01627T-099TOPM-099Y-099M-099Y-16M-0WGY |
| 447 | ZINCOL/5/2*QUAIU #1/3/T.DICOCCON PI94625/AE.SQUARROSA (372)//3*PASTOR/4/QUAIU #2 | CMSS13Y01630T-099TOPM-099Y-099M-099Y-13M-0WGY |
| 448 | WHEAR/KIRITATI/3/C80.1/3*BATAVIA//2*WBLL1/4/CMH75A.66/SERI/5/2*VILLA JUAREZ F2009/3/T.DICOCCON PI94625/AE.SQUARROSA (372)//3*PASTOR/4/WBLL1*2/BRAMBLING | CMSS13Y01643T-099TOPM-099Y-099M-099Y-1M-0WGY |
| 449 | PAURAQ//RL6043/4*NAC/3/2*QUAIU #1/SOLALA//QUAIU #2 | CMSS13Y01652T-099TOPM-099Y-099M-099Y-3M-0WGY |
| 450 | PAURAQ//AG/5*NAC/3/2*QUAIU #1/SOLALA//QUAIU #2 | CMSS13Y01654T-099TOPM-099Y-099M-099Y-23M-0WGY |
