# Supplementary material for: Variation in Grain Zinc and Iron Concentrations, Grain Yield and Associated Traits of Biofortified Bread Wheat Genotypes in Nepal
Source: Front Plant Sci. 2022 Jun 13;13:881965. doi: 10.3389/fpls.2022.881965 (PMC9249123; doi:10.3389/fpls.2022.881965)
Supplement: Supplementary file 2 [file Data_Sheet_2.docx]

Supplementary Material

**Supplementary Table 2 |** Performance of different genotypes tested in the 6^th^ HarvestPlus Yield Trial during 2015–16

| **Entry No.** | **DTH** | **DTM** | **Pht (cm)** | **TGW (gm)** | **GY (kg/ha)** | **GrainFe**  **(ppm)** | **GrainZn**  **(ppm)** |
| --- | --- | --- | --- | --- | --- | --- | --- |
| 401 | 113 | 174 | 83 | 44.8 | 3322 | 41.8 | 33.3 |
| 402 | 107 | 162 | 82 | 47.8 | 3283 | 40.5 | 34.2 |
| 403 | 112 | 165 | 83 | 48.6 | 3292 | 41.9 | 34.1 |
| 404 | 108 | 161 | 89 | 47.4 | 3237 | 44.8 | 36.3 |
| 405 | 114 | 165 | 92 | 48.5 | 3172 | 42.6 | 35.5 |
| 406 | 111 | 165 | 88 | 50.6 | 3352 | 42.7 | 33.9 |
| 407 | 110 | 168 | 93 | 49.1 | 3306 | 42.5 | 34.3 |
| 408 | 110 | 166 | 92 | 51.5 | 3308 | 42.0 | 35.2 |
| 409 | 112 | 167 | 88 | 44.1 | 3319 | 42.4 | 34.6 |
| 410 | 111 | 166 | 94 | 52.3 | 3318 | 42.2 | 34.9 |
| 411 | 113 | 166 | 90 | 51.1 | 3337 | 41.2 | 34.8 |
| 412 | 111 | 167 | 90 | 43.4 | 3245 | 40.7 | 34.5 |
| 413 | 109 | 165 | 89 | 41.2 | 3337 | 41.1 | 34.2 |
| 414 | 109 | 163 | 91 | 42.8 | 3299 | 40.3 | 33.9 |
| 415 | 108 | 163 | 91 | 52.0 | 3352 | 42.0 | 33.5 |
| 416 | 110 | 165 | 88 | 45.9 | 3326 | 41.0 | 34.1 |
| 417 | 110 | 165 | 93 | 47.7 | 3298 | 39.6 | 33.7 |
| 418 | 109 | 163 | 83 | 49.0 | 3290 | 41.2 | 34.9 |
| 419 | 113 | 166 | 86 | 45.0 | 3329 | 41.5 | 34.9 |
| 420 | 113 | 165 | 85 | 48.4 | 3288 | 41.5 | 33.6 |
| 421 | 111 | 163 | 85 | 46.2 | 3281 | 43.6 | 35.0 |
| 422 | 112 | 166 | 90 | 50.3 | 3335 | 40.7 | 33.8 |
| 423 | 108 | 164 | 91 | 48.6 | 3342 | 40.8 | 34.6 |
| 424 | 110 | 165 | 91 | 45.9 | 3322 | 41.8 | 34.5 |
| 425 | 111 | 165 | 91 | 48.2 | 3326 | 42.9 | 34.6 |
| 426 | 108 | 164 | 87 | 41.0 | 3289 | 42.8 | 33.8 |
| 427 | 111 | 165 | 90 | 47.6 | 3323 | 40.8 | 34.0 |
| 428 | 109 | 165 | 92 | 44.3 | 3307 | 41.5 | 33.7 |
| 429 | 112 | 166 | 87 | 46.8 | 3307 | 41.0 | 34.3 |
| 430 | 111 | 166 | 89 | 47.9 | 3342 | 41.3 | 33.6 |
| 431 | 111 | 165 | 90 | 53.8 | 3420 | 41.9 | 34.3 |
| 432 | 110 | 166 | 91 | 45.0 | 3342 | 42.4 | 35.2 |
| 433 | 111 | 166 | 91 | 54.8 | 3370 | 41.4 | 33.4 |
| 434 | 113 | 167 | 90 | 41.8 | 3309 | 40.9 | 34.3 |
| 435 | 108 | 164 | 91 | 48.0 | 3331 | 40.7 | 34.5 |
| 436 | 112 | 168 | 88 | 42.6 | 3271 | 40.5 | 35.2 |
| 437 | 109 | 165 | 87 | 49.5 | 3423 | 40.8 | 33.6 |
| 438 | 109 | 164 | 91 | 50.0 | 3316 | 41.2 | 34.2 |
| 439 | 110 | 164 | 87 | 47.0 | 3303 | 42.1 | 35.5 |
| 440 | 109 | 164 | 86 | 45.6 | 3266 | 43.2 | 35.4 |
| 441 | 110 | 164 | 94 | 43.9 | 3293 | 40.9 | 33.8 |
| 442 | 108 | 164 | 91 | 49.9 | 3345 | 41.3 | 34.6 |
| 443 | 110 | 166 | 90 | 47.2 | 3407 | 42.6 | 33.4 |
| 444 | 109 | 164 | 91 | 52.6 | 3343 | 41.8 | 33.6 |
| 445 | 110 | 165 | 91 | 50.0 | 3320 | 43.3 | 35.0 |
| 446 | 111 | 166 | 88 | 46.3 | 3391 | 41.4 | 34.7 |
| 447 | 110 | 163 | 91 | 47.7 | 3382 | 42.7 | 35.3 |
| 448 | 112 | 167 | 90 | 46.8 | 3342 | 41.4 | 34.2 |
| 449 | 110 | 166 | 92 | 46.0 | 3244 | 41.5 | 34.6 |
| 450 | 112 | 166 | 91 | 52.0 | 3291 | 42.5 | 34.7 |
| **Kabre** | 108 | 158 | 81 | 50.5 | 1887 | 43.7 | 34.2 |
| **Khumaltar** | 131 | 173 | 94 | 46.1 | 4506 | 46.7 | 48.7 |
| **Surkhet** | 91 | X | 92 | 46.1 | 3560 | 34.7 | 20.3 |
| **Mean** | **110** | **165** | **89** | **47.6** | **3318** | **41.7** | **34.4** |
| **p-level G** | 0.000 | 0.000 | 0.000 | 0.000 | 0.475 | 0.062 | 0.255 |
| **p-level Loc** | 0.000 | 0.000 | 0.000 | 0.000 | 0.000 | 0.000 | 0.000 |

DTH: days to heading; DTM: days to maturity; Pht: plant height; TGW: thousand grain weight; GY: grain yield; GrainFe: grain iron content; GrainZn: grain zinc content

**Supplementary Table 3 |** Performance of different genotypes tested in the 7^th^ HarvestPlus Yield Trial during 2016–17

| **Entry No.** | **DTH** | **DTM** | **Pht (cm)** | **Tillers** | **GNS** | **GWS**  **(gm)** | **TGW**  **(gm)** | **GY (kg/ha)** | **GrainFe**  **(ppm)** | **GrainZn**  **(ppm)** |
| --- | --- | --- | --- | --- | --- | --- | --- | --- | --- | --- |
| 401 | 107 | 152 | 92 | 281 | 50 | 2.7 | 53.5 | 3861 | 42.7 | 33.0 |
| 402 | 100 | 146 | 88 | 277 | 52 | 2.7 | 48.8 | 4048 | 43.8 | 33.3 |
| 403 | 104 | 151 | 90 | 269 | 56 | 2.8 | 48.3 | 3744 | 44.3 | 32.4 |
| 404 | 100 | 144 | 92 | 270 | 46 | 2.5 | 52.7 | 3495 | 47.5 | 38.1 |
| 405 | 99 | 149 | 92 | 270 | 57 | 2.9 | 48.4 | 3993 | 43.3 | 32.6 |
| 406 | 101 | 150 | 91 | 233 | 52 | 2.9 | 53.3 | 3671 | 44.9 | 32.8 |
| 407 | 104 | 149 | 89 | 252 | 53 | 3.1 | 57.9 | 3769 | 45.1 | 34.0 |
| 408 | 105 | 149 | 91 | 261 | 51 | 2.7 | 53.9 | 3809 | 44.0 | 33.3 |
| 409 | 104 | 149 | 90 | 277 | 52 | 2.7 | 48.6 | 3721 | 45.9 | 34.7 |
| 410 | 107 | 153 | 90 | 281 | 56 | 2.9 | 51.7 | 3910 | 44.1 | 33.3 |
| 411 | 107 | 152 | 88 | 261 | 54 | 2.9 | 53.5 | 4036 | 44.7 | 32.7 |
| 412 | 108 | 154 | 88 | 275 | 56 | 2.8 | 49.3 | 3942 | 43.2 | 33.7 |
| 413 | 102 | 148 | 92 | 247 | 52 | 2.8 | 53.7 | 3599 | 42.2 | 32.5 |
| 414 | 103 | 150 | 92 | 285 | 51 | 2.6 | 49.4 | 3884 | 42.9 | 32.6 |
| 415 | 105 | 150 | 95 | 257 | 52 | 2.8 | 53.0 | 3748 | 43.7 | 32.4 |
| 416 | 101 | 149 | 91 | 261 | 49 | 2.7 | 52.5 | 3730 | 42.2 | 32.2 |
| 417 | 103 | 149 | 92 | 255 | 56 | 2.9 | 50.7 | 3870 | 45.4 | 33.4 |
| 418 | 101 | 148 | 88 | 252 | 48 | 2.7 | 55.7 | 3629 | 48.1 | 34.6 |
| 419 | 100 | 148 | 87 | 229 | 50 | 2.8 | 53.7 | 3310 | 44.6 | 34.3 |
| 420 | 103 | 148 | 92 | 251 | 52 | 2.7 | 50.7 | 3600 | 43.7 | 35.7 |
| 421 | 101 | 149 | 92 | 262 | 53 | 2.8 | 50.3 | 3795 | 44.6 | 34.2 |
| 422 | 105 | 150 | 91 | 265 | 56 | 2.9 | 51.4 | 3725 | 43.9 | 33.8 |
| 423 | 100 | 149 | 92 | 259 | 51 | 2.8 | 54.4 | 3842 | 45.4 | 33.5 |
| 424 | 106 | 151 | 89 | 278 | 55 | 2.8 | 50.1 | 3957 | 43.0 | 34.1 |
| 425 | 104 | 151 | 89 | 253 | 52 | 2.9 | 54.5 | 3615 | 46.0 | 34.5 |
| 426 | 106 | 152 | 96 | 259 | 54 | 2.8 | 54.3 | 3872 | 44.1 | 33.1 |
| 427 | 104 | 150 | 90 | 253 | 50 | 2.7 | 53.1 | 3772 | 42.9 | 34.0 |
| 428 | 101 | 149 | 89 | 262 | 51 | 2.7 | 48.5 | 3606 | 44.1 | 33.0 |
| 429 | 103 | 152 | 90 | 271 | 50 | 2.7 | 49.6 | 4026 | 45.7 | 35.5 |
| 430 | 103 | 151 | 90 | 283 | 51 | 2.7 | 48.7 | 3827 | 45.0 | 32.9 |
| 431 | 108 | 153 | 86 | 264 | 52 | 2.8 | 51.1 | 3569 | 45.7 | 31.8 |
| 432 | 102 | 147 | 90 | 255 | 53 | 2.8 | 51.5 | 3567 | 45.0 | 34.6 |
| 433 | 104 | 151 | 88 | 253 | 52 | 2.8 | 51.4 | 3739 | 44.4 | 34.0 |
| 434 | 104 | 151 | 89 | 271 | 53 | 2.7 | 50.0 | 3648 | 43.8 | 33.5 |
| 435 | 102 | 149 | 93 | 264 | 52 | 2.8 | 52.0 | 3912 | 43.6 | 32.2 |
| 436 | 104 | 150 | 91 | 248 | 56 | 3.0 | 50.8 | 3699 | 45.0 | 33.4 |
| 437 | 103 | 150 | 91 | 268 | 55 | 2.7 | 47.1 | 3889 | 44.2 | 34.4 |
| 438 | 100 | 147 | 94 | 262 | 51 | 2.7 | 50.8 | 3665 | 43.1 | 33.5 |
| 439 | 105 | 151 | 89 | 266 | 52 | 2.7 | 46.8 | 3597 | 43.4 | 35.0 |
| 440 | 104 | 150 | 95 | 263 | 50 | 3.0 | 56.4 | 3749 | 44.2 | 34.9 |
| 441 | 101 | 149 | 92 | 252 | 53 | 2.8 | 52.3 | 4022 | 44.2 | 34.5 |
| 442 | 103 | 151 | 92 | 267 | 50 | 2.7 | 53.1 | 3830 | 44.4 | 33.7 |
| 443 | 107 | 151 | 94 | 267 | 55 | 2.8 | 48.8 | 3810 | 43.1 | 34.1 |
| 444 | 106 | 153 | 89 | 249 | 54 | 3.1 | 54.8 | 3624 | 42.9 | 34.3 |
| 445 | 107 | 152 | 90 | 268 | 53 | 2.8 | 50.4 | 3993 | 42.5 | 33.5 |
| 446 | 106 | 152 | 91 | 279 | 55 | 2.9 | 52.1 | 4016 | 42.1 | 32.9 |
| 447 | 106 | 152 | 89 | 261 | 56 | 2.9 | 49.5 | 3975 | 43.7 | 33.2 |
| 448 | 102 | 150 | 90 | 274 | 50 | 2.7 | 51.4 | 3916 | 44.0 | 33.0 |
| 449 | 107 | 152 | 93 | 252 | 53 | 2.9 | 55.7 | 3840 | 42.9 | 34.5 |
| 450 | 102 | 149 | 92 | 265 | 52 | 2.7 | 49.9 | 3832 | 44.1 | 33.6 |
| **Doti** | 93 | 134 | 95 | X | X | X | 46.3 | 2616.5 | 53.7 | 35.8 |
| **Kabre** | 111 | 169 | 78 | 222.7 | X | X | 53.0 | 3189.8 | 44.6 | 31.5 |
| **Khumaltar** | 126 | 174 | 104 | 310.6 | 58.5 | 3.1 | 52.9 | 6527.7 | 40.8 | 43.9 |
| **Surkhet** | 84 | 123 | 87 | 254.6 | 46.4 | 2.5 | 54.2 | 2809.4 | 37.5 | 23.5 |
| **Mean** | 104 | 150 | 91 | 262.6 | 52.5 | 2.8 | 51.6 | 3785.8 | 44.1 | 33.7 |
| **p-level G** | 0.000 | 0.000 | 0.000 | 0.003 | 0.004 | 0.031 | 0.000 | 0.004 | 0.025 | 0.016 |
| **p-level Loc** | 0.000 | 0.000 | 0.000 | 0.000 | 0.000 | 0.000 | 0.000 | 0.000 | 0.000 | 0.000 |

DTH: days to heading; DTM: days to maturity; Pht: plant height; GNS: grain number per spike; GWS: grain weight per spike; TGW: thousand grain weight; GY: grain yield; GrainFe: grain iron content; GrainZn: grain zinc content

**Supplementary Table 4 |** Performance of different genotypes tested in the 8^th^ HarvestPlus Yield Trial during 2017–18

| **Entry No.** | **DTH** | **DTM** | **Pht (cm)** | **SpkL** | **Tillers** | **GNS** | **TGW (gm)** | **GY (kg/ha)** | **GrainFe**  **(ppm)** | **GrainZn**  **(ppm)** |
| --- | --- | --- | --- | --- | --- | --- | --- | --- | --- | --- |
| 401 | 110 | 153 | 81 | 9.6 | 242 | 53 | 48.2 | 3258 | 38.4 | 28.3 |
| 402 | 103 | 145 | 84 | 9.1 | 227 | 53 | 47.8 | 3606 | 41.4 | 28.8 |
| 403 | 107 | 149 | 81 | 9.1 | 195 | 53 | 44.5 | 3340 | 38.3 | 28.3 |
| 404 | 100 | 141 | 85 | 9.2 | 208 | 53 | 48.8 | 2865 | 41.1 | 30.7 |
| 405 | 105 | 147 | 80 | 9.3 | 218 | 53 | 46.4 | 3803 | 39.8 | 28.7 |
| 406 | 103 | 142 | 88 | 9.0 | 197 | 53 | 51.1 | 3569 | 40.2 | 29.7 |
| 407 | 106 | 149 | 79 | 9.5 | 197 | 53 | 48.8 | 3374 | 40.6 | 30.0 |
| 408 | 108 | 150 | 80 | 10.0 | 208 | 53 | 48.8 | 3233 | 39.6 | 28.5 |
| 409 | 104 | 148 | 84 | 9.5 | 210 | 53 | 46.1 | 3331 | 38.1 | 29.4 |
| 410 | 106 | 150 | 86 | 9.7 | 187 | 53 | 49.3 | 3378 | 40.4 | 30.5 |
| 411 | 107 | 150 | 83 | 9.1 | 173 | 53 | 47.1 | 2866 | 39.6 | 30.0 |
| 412 | 105 | 148 | 84 | 9.4 | 202 | 53 | 51.6 | 3617 | 38.7 | 31.0 |
| 413 | 105 | 148 | 87 | 9.5 | 196 | 53 | 50.8 | 3250 | 39.8 | 28.8 |
| 414 | 106 | 150 | 86 | 9.3 | 210 | 53 | 50.3 | 3570 | 38.7 | 28.9 |
| 415 | 108 | 149 | 84 | 9.7 | 216 | 53 | 46.7 | 3490 | 39.7 | 29.3 |
| 416 | 104 | 148 | 87 | 9.4 | 204 | 53 | 49.7 | 3863 | 41.4 | 30.2 |
| 417 | 108 | 152 | 82 | 9.7 | 193 | 53 | 46.4 | 2829 | 49.2 | 31.8 |
| 418 | 105 | 148 | 86 | 9.5 | 199 | 53 | 49.4 | 3183 | 42.2 | 28.5 |
| 419 | 107 | 149 | 84 | 9.7 | 185 | 53 | 49.0 | 3342 | 40.9 | 29.5 |
| 420 | 104 | 145 | 84 | 9.7 | 198 | 53 | 49.9 | 3397 | 39.9 | 29.5 |
| 421 | 104 | 148 | 88 | 9.6 | 205 | 53 | 44.2 | 3438 | 38.8 | 28.2 |
| 422 | 104 | 147 | 86 | 9.2 | 189 | 53 | 48.8 | 3155 | 39.6 | 28.9 |
| 423 | 106 | 149 | 85 | 9.8 | 191 | 53 | 49.3 | 3224 | 40.2 | 30.0 |
| 424 | 103 | 147 | 84 | 9.4 | 196 | 53 | 48.9 | 3274 | 40.2 | 28.7 |
| 425 | 109 | 150 | 81 | 9.4 | 199 | 53 | 47.6 | 3043 | 39.8 | 30.8 |
| 426 | 106 | 149 | 82 | 9.6 | 197 | 53 | 49.5 | 3178 | 39.6 | 28.6 |
| 427 | 107 | 151 | 85 | 9.4 | 207 | 53 | 51.7 | 3443 | 38.9 | 30.1 |
| 428 | 106 | 152 | 83 | 9.4 | 224 | 54 | 45.0 | 3224 | 41.6 | 28.6 |
| 429 | 103 | 147 | 88 | 9.4 | 209 | 53 | 51.9 | 3670 | 40.0 | 28.8 |
| 430 | 105 | 148 | 87 | 9.8 | 203 | 53 | 47.5 | 3583 | 39.4 | 28.3 |
| 431 | 107 | 151 | 81 | 9.5 | 192 | 53 | 54.3 | 3321 | 38.7 | 27.5 |
| 432 | 109 | 153 | 86 | 9.1 | 207 | 53 | 44.9 | 3316 | 39.2 | 29.6 |
| 433 | 109 | 152 | 78 | 9.3 | 181 | 53 | 48.0 | 2959 | 40.5 | 29.6 |
| 434 | 107 | 150 | 83 | 9.2 | 190 | 53 | 45.8 | 3185 | 39.6 | 28.4 |
| 435 | 103 | 145 | 85 | 9.6 | 187 | 53 | 50.4 | 3166 | 42.2 | 30.9 |
| 436 | 106 | 148 | 81 | 9.4 | 179 | 53 | 50.0 | 3008 | 39.4 | 29.0 |
| 437 | 108 | 151 | 84 | 9.2 | 199 | 53 | 47.1 | 3090 | 39.4 | 29.1 |
| 438 | 111 | 152 | 81 | 9.1 | 186 | 53 | 47.3 | 2942 | 40.2 | 29.3 |
| 439 | 110 | 149 | 80 | 9.4 | 192 | 53 | 43.5 | 3162 | 38.8 | 29.9 |
| 440 | 109 | 152 | 75 | 9.4 | 191 | 53 | 46.5 | 3203 | 37.6 | 30.0 |
| 441 | 108 | 150 | 76 | 9.5 | 206 | 53 | 46.2 | 2985 | 39.7 | 29.7 |
| 442 | 104 | 149 | 81 | 9.4 | 188 | 53 | 48.0 | 2988 | 41.2 | 29.5 |
| 443 | 104 | 144 | 88 | 9.1 | 181 | 53 | 48.8 | 3123 | 43.4 | 31.1 |
| 444 | 106 | 149 | 78 | 9.4 | 180 | 53 | 46.6 | 2870 | 39.0 | 28.9 |
| 445 | 107 | 152 | 81 | 9.9 | 181 | 53 | 45.3 | 3347 | 39.4 | 28.6 |
| 446 | 111 | 153 | 83 | 9.4 | 194 | 53 | 46.4 | 3242 | 38.1 | 28.7 |
| 447 | 107 | 151 | 83 | 9.5 | 188 | 53 | 45.4 | 3237 | 39.3 | 28.2 |
| 448 | 107 | 151 | 80 | 9.8 | 193 | 53 | 47.0 | 3299 | 39.2 | 29.9 |
| 449 | 105 | 148 | 84 | 9.2 | 207 | 53 | 48.4 | 3593 | 37.9 | 28.8 |
| 450 | 106 | 148 | 101 | 9.2 | 180 | 53 | 46.2 | 3117 | 39.7 | 28.8 |
| **Doti** | 96 | 136 | 84 | 10.3 | 192 | 47 | 47.7 | 3801 | 48.1 | 26.0 |
| **Kabre** | 108 | 150 | 68 | 6.4 | 196 | 0 | 47.5 | 2113 | 36.3 | 20.1 |
| **Khumaltar** | 114 | 161 | 98 | 11.5 | 206 | 59 | 48.9 | 3898 | 40.1 | 42.0 |
| **Bhairahawa** | X | X | X | X | X | X | X | X | 32.8 | 29.2 |
| **Nepalgunj** | X | X | X | X | X | X | X | X | 42.5 | 29.4 |
| **Mean** | 106 | 149 | 83 | 9.4 | 198.0 | 53.3 | 48.0 | 3271.0 | 40.0 | 29.3 |
| **p-level G** | 0.000 | 0.000 | 0.802 | 0.029 | 0.006 | 0.856 | 0.000 | 0.001 | 0.000 | 0.002 |
| **p-level Loc** | 0.000 | 0.000 | 0.000 | 0.000 | 0.145 | < 2e-16 | 0.006 | 0.000 | 0.000 | 0.000 |

DTH: days to heading; DTM: days to maturity; Pht: plant height; SpkL: spike length; GNS: grain number per spike; TGW: thousand grain weight; GY: grain yield; GrainFe: grain iron content; GrainZn: grain zinc content

**Supplementary Table 5 |** Performance of different genotypes tested in the 9^th^ HarvestPlus Yield Trial during 2018–19

| **Entry No.** | **DTH** | **DTM** | **Pht (cm)** | **Tillers** | **GNS** | **GWS**  **(gm)** | **TGW**  **(gm)** | **GY (kg/ha)** | **GrainFe**  **(ppm)** | **GrainZn**  **(ppm)** |
| --- | --- | --- | --- | --- | --- | --- | --- | --- | --- | --- |
| 401 | 103 | 147 | 87 | 373 | 57 | 2.47 | 43.7 | 4675 | 30.1 | 28.5 |
| 402 | 102 | 145 | 83 | 367 | 55 | 2.46 | 43.1 | 4544 | 31.0 | 27.6 |
| 403 | 100 | 144 | 84 | 371 | 60 | 2.61 | 43.6 | 4507 | 33.2 | 29.2 |
| 404 | 93 | 139 | 88 | 365 | 48 | 2.30 | 43.8 | 4460 | 36.6 | 33.5 |
| 405 | 100 | 144 | 93 | 355 | 55 | 2.52 | 44.1 | 4656 | 32.0 | 30.2 |
| 406 | 103 | 145 | 87 | 364 | 59 | 2.56 | 43.5 | 4716 | 33.3 | 28.0 |
| 407 | 102 | 145 | 88 | 354 | 56 | 2.50 | 43.1 | 4544 | 32.3 | 31.1 |
| 408 | 105 | 144 | 88 | 356 | 58 | 2.58 | 43.5 | 4566 | 31.8 | 30.3 |
| 409 | 101 | 144 | 87 | 343 | 57 | 2.57 | 44.3 | 4549 | 30.4 | 32.5 |
| 410 | 103 | 142 | 86 | 359 | 52 | 2.47 | 44.5 | 4716 | 32.1 | 31.8 |
| 411 | 104 | 144 | 85 | 351 | 52 | 2.35 | 43.9 | 4630 | 35.0 | 32.7 |
| 412 | 102 | 145 | 88 | 352 | 60 | 2.68 | 44.2 | 4595 | 32.5 | 30.4 |
| 413 | 99 | 145 | 89 | 359 | 57 | 2.67 | 44.9 | 4678 | 32.4 | 30.5 |
| 414 | 102 | 144 | 87 | 352 | 55 | 2.57 | 44.4 | 4542 | 32.1 | 31.1 |
| 415 | 103 | 145 | 87 | 356 | 54 | 2.46 | 44.2 | 4607 | 32.1 | 30.8 |
| 416 | 100 | 144 | 88 | 358 | 55 | 2.55 | 44.7 | 4627 | 35.7 | 30.7 |
| 417 | 101 | 145 | 85 | 354 | 58 | 2.66 | 44.5 | 4559 | 33.6 | 30.1 |
| 418 | 101 | 145 | 88 | 356 | 57 | 2.64 | 44.8 | 4559 | 32.0 | 28.6 |
| 419 | 102 | 144 | 84 | 349 | 56 | 2.59 | 44.2 | 4670 | 35.8 | 32.9 |
| 420 | 101 | 145 | 84 | 360 | 57 | 2.52 | 44.0 | 4509 | 34.3 | 29.3 |
| 421 | 100 | 144 | 87 | 363 | 55 | 2.43 | 43.1 | 4724 | 32.1 | 29.2 |
| 422 | 102 | 144 | 89 | 370 | 56 | 2.50 | 42.8 | 4647 | 32.0 | 30.9 |
| 423 | 103 | 144 | 85 | 358 | 54 | 2.61 | 50.1 | 4553 | 33.3 | 29.6 |
| 424 | 102 | 144 | 87 | 366 | 55 | 2.54 | 44.0 | 4607 | 33.0 | 31.0 |
| 425 | 101 | 143 | 84 | 368 | 56 | 2.54 | 43.6 | 4500 | 31.5 | 29.3 |
| 426 | 101 | 146 | 84 | 368 | 55 | 2.49 | 43.7 | 4636 | 31.4 | 29.6 |
| 427 | 97 | 141 | 87 | 365 | 53 | 2.45 | 43.7 | 4635 | 33.3 | 31.1 |
| 428 | 103 | 143 | 84 | 358 | 50 | 2.45 | 44.6 | 4474 | 33.8 | 31.5 |
| 429 | 101 | 144 | 88 | 364 | 60 | 2.49 | 42.2 | 4577 | 33.7 | 29.8 |
| 430 | 98 | 142 | 83 | 347 | 52 | 2.51 | 44.2 | 4545 | 32.9 | 29.4 |
| 431 | 103 | 146 | 85 | 365 | 57 | 2.48 | 43.1 | 4757 | 31.1 | 28.6 |
| 432 | 99 | 142 | 87 | 357 | 58 | 2.65 | 44.3 | 4542 | 35.0 | 30.0 |
| 433 | 97 | 142 | 88 | 355 | 56 | 2.48 | 43.0 | 4710 | 34.0 | 31.2 |
| 434 | 102 | 145 | 89 | 359 | 58 | 2.56 | 43.7 | 4619 | 34.6 | 30.6 |
| 435 | 103 | 145 | 89 | 347 | 56 | 2.60 | 44.1 | 4464 | 31.8 | 30.5 |
| 436 | 103 | 145 | 84 | 352 | 54 | 2.58 | 43.4 | 4337 | 30.8 | 29.1 |
| 437 | 102 | 144 | 84 | 363 | 55 | 2.50 | 43.7 | 4459 | 32.9 | 30.3 |
| 438 | 102 | 145 | 86 | 342 | 58 | 2.59 | 43.6 | 4526 | 31.8 | 30.5 |
| 439 | 103 | 145 | 86 | 355 | 57 | 2.51 | 43.1 | 4551 | 32.0 | 30.5 |
| 440 | 101 | 144 | 88 | 362 | 55 | 2.57 | 44.4 | 4775 | 32.2 | 28.9 |
| 441 | 100 | 143 | 82 | 356 | 59 | 2.58 | 43.6 | 4685 | 32.4 | 30.3 |
| 442 | 98 | 143 | 86 | 355 | 54 | 2.53 | 44.5 | 4521 | 36.0 | 28.6 |
| 443 | 101 | 144 | 89 | 364 | 59 | 2.66 | 43.6 | 4571 | 32.3 | 28.8 |
| 444 | 102 | 144 | 87 | 339 | 57 | 2.61 | 43.8 | 4536 | 32.7 | 29.3 |
| 445 | 101 | 144 | 86 | 363 | 58 | 2.60 | 43.9 | 4686 | 32.3 | 31.3 |
| 446 | 101 | 145 | 91 | 354 | 57 | 2.63 | 44.8 | 4667 | 32.5 | 28.3 |
| 447 | 100 | 143 | 89 | 372 | 55 | 2.57 | 45.0 | 4711 | 30.9 | 29.3 |
| 448 | 102 | 143 | 82 | 360 | 50 | 2.49 | 44.7 | 4697 | 33.2 | 33.0 |
| 449 | 100 | 144 | 89 | 371 | 53 | 2.49 | 43.8 | 4636 | 31.3 | 30.3 |
| 450 | 100 | 145 | 90 | 367 | 56 | 2.54 | 44.0 | 4757 | 31.3 | 30.7 |
| **Bhairahawa** | 86 | 119 | 92 |  |  |  | 41.5 | 4169.7 | 34.0 | 35.8 |
| **Doti** | 101 | 143 | 89 |  |  |  | 42.0 | 4207.1 | 31.3 | 19.1 |
| **Kabre** | 105 | 149 | 72 | 254 | 48.2 | 2.39 | 49.2 | 3555.6 | 34.1 | 28.6 |
| **Khumaltar** | 121 | 165 | 103 | 464 | 63.1 | 2.69 | 43.0 | 6239.6 | 34.6 | 48.1 |
| **Surkhet** | 93 |  | 77 |  |  |  | 44.4 | 4829.4 | 29.7 | 19.5 |
| **Mean** | 101.1 | 144.0 | 86.6 | 358.8 | 55.7 | 2.54 | 44.0 | 4600.3 | 32.7 | 30.2 |
| **p-level G** | 0.000 | 0.000 | 0.000 | 0.368 | 0.002 | 0.081 | 0.199 | 0.222 | 0.000 | 0.000 |
| **p-level Loc** | 0.000 | 0.000 | 0.000 | 0.000 | 0.000 | 0.000 | 0.000 | 0.000 | 0.000 | 0.000 |

DTH: days to heading; DTM: days to maturity; Pht: plant height; GNS: grain number per spike; GWS: grain weight per spike; TGW: thousand grain weight; GY: grain yield; GrainFe: grain iron content; GrainZn: grain zinc content
